# Two Novel DNAs That Enhance Symptoms and Overcome CMD2 Resistance to Cassava Mosaic Disease

**DOI:** 10.1128/JVI.02834-15

**Published:** 2016-03-28

**Authors:** Joseph Ndunguru, Leandro De León, Catherine D. Doyle, Peter Sseruwagi, German Plata, James P. Legg, Graham Thompson, Joe Tohme, Theresa Aveling, Jose T. Ascencio-Ibáñez, Linda Hanley-Bowdoin

**Affiliations:** a Mikocheni Agricultural Research Institute, Dar es Salaam, Tanzania; b Department of Molecular and Structural Biochemistry, North Carolina State University, Raleigh, North Carolina, USA; c Department of Plant and Microbial Biology, North Carolina State University, Raleigh, North Carolina, USA; d Center for Computational Biology and Bioinformatics, Columbia University, New York, New York, USA; e International Institute of Tropical Agriculture-Tanzania, Dar es Salaam, Tanzania; f ARC-Institute for Industrial Crops, Rusternburg, South Africa; g International Center for Tropical Agriculture, Cali, Colombia; h University of Pretoria, Department of Microbiology and Plant Pathology, Pretoria, South Africa; University of Maryland

## Abstract

Cassava mosaic begomoviruses (CMBs) cause cassava mosaic disease (CMD) across Africa and the Indian subcontinent. Like all members of the geminivirus family, CMBs have small, circular single-stranded DNA genomes. We report here the discovery of two novel DNA sequences, designated SEGS-1 and SEGS-2 (for sequences enhancing geminivirus symptoms), that enhance symptoms and break resistance to CMD. The SEGS are characterized by GC-rich regions and the absence of long open reading frames. Both SEGS enhanced CMD symptoms in cassava (Manihot esculenta Crantz) when coinoculated with *African cassava mosaic virus* (ACMV), *East African cassava mosaic Cameroon virus* (EACMCV), or *East African cassava mosaic virus-Uganda* (EACMV-UG). SEGS-1 also overcame resistance of a cassava landrace carrying the CMD2 resistance locus when coinoculated with EACMV-UG. Episomal forms of both SEGS were detected in CMB-infected cassava but not in healthy cassava. SEGS-2 episomes were also found in virions and whiteflies. SEGS-1 has no homology to geminiviruses or their associated satellites, but the cassava genome contains a sequence that is 99% identical to full-length SEGS-1. The cassava genome also includes three sequences with 84 to 89% identity to SEGS-2 that together encompass all of SEGS-2 except for a 52-bp region, which includes the episomal junction and a 26-bp sequence related to alphasatellite replication origins. These results suggest that SEGS-1 is derived from the cassava genome and facilitates CMB infection as an integrated copy and/or an episome, while SEGS-2 was originally from the cassava genome but now is encapsidated into virions and transmitted as an episome by whiteflies.

**IMPORTANCE** Cassava is a major crop in the developing world, with its production in Africa being second only to maize. CMD is one of the most important diseases of cassava and a serious constraint to production across Africa. CMD2 is a major CMD resistance locus that has been deployed in many cassava cultivars through large-scale breeding programs. In recent years, severe, atypical CMD symptoms have been observed occasionally on resistant cultivars, some of which carry the CMD2 locus, in African fields. In this report, we identified and characterized two DNA sequences, SEGS-1 and SEGS-2, which produce similar symptoms when coinoculated with cassava mosaic begomoviruses onto a susceptible cultivar or a CMD2-resistant landrace. The ability of SEGS-1 to overcome CMD2 resistance and the transmission of SEGS-2 by whiteflies has major implications for the long-term durability of CMD2 resistance and underscore the need for alternative sources of resistance in cassava.

## INTRODUCTION

Cassava (Manihot esculenta Crantz) is an important root crop in Africa and Asia, where it is eaten by ca. 400 million people every day (1). Cassava can grow under drought, high-temperature, and poor soil conditions, but its production is severely limited by viral diseases (2). Cassava mosaic disease (CMD) is one of the most economically important crop diseases in Africa (3). Extensive efforts to develop CMD-resistant cassava led to the discovery of the CMD2 resistance locus in the Nigerian landrace, TME3, and its widespread introgression into other cassava cultivars (4, 5). Recently, CMD2 was mapped to a single sequence scaffold in the cassava genome (6). Some cultivars carrying the CMD2 locus are nearly immune, while others have reduced viral titer and attenuated symptoms, indicating that genetic background influences CMD2 resistance (4).

CMD is caused by 9 geminivirus species, which collectively are designated cassava mosaic begomoviruses (CMBs) and comprise more than 27 strains in Africa. CMBs often occur in mixed infections and undergo reassortment to form pseudorecombinants and/or recombination to generate new chimeric viral DNA components (7–12). They also display high mutation rates (13). The resulting variation has been associated with the emergence of new viruses with altered virulence (9) and a severe pandemic in the 1990s and 2000s (14).

Begomoviruses are transmitted by whiteflies (Bemisia tabaci Genn.) and occur in two lineages, the Old World viruses and the New World viruses (15). They have single-stranded DNA (ssDNA) genomes that can occur as one or two components. CMBs have bipartite genomes consisting of DNA-A and DNA-B (16), which together encode 8 proteins necessary for viral replication, transcription, movement, and encapsidation as well as for countering host defenses (17–22). Both CMB DNA components are essential for infection and contain a conserved intergenic region that includes the viral promoters and the replication origin (23, 24). Like all geminiviruses, CMBs replicate through double-stranded DNA (dsDNA) intermediates in the nuclei of infected plant cells (25) and recruit host proteins for their replication, expression, and movement in plants (26).

Episomal, circular ssDNAs termed alphasatellites and betasatellites are associated with several begomovirus species (for a review, see reference 27) and, more recently, with a mastrevirus (28, 29). The satellites first were reported in association with *Cotton leaf curl Multan virus* (30) and *Ageratum yellow vein virus* (31) and are approximately half the size of their helper virus genomic components. Since their discovery, these satellites have been found with many monopartite begomoviruses (32, 33) and a few bipartite begomoviruses (34, 35). Recently, an alphasatellite was found in association with a CMB in Madagascar (36). A third type of episome linked to begomoviruses has been found in tomato and malvaceous plants (37, 38). These molecules share homology with betasatellites but are half the size and lack detectable open reading frames.

Betasatellites require the replication protein (Rep) of their helper begomovirus to amplify their DNAs, while alphasatellites encode their own Rep and replicate autonomously (for a review, see reference 39). Both satellite DNAs are encapsidated into virions and transmitted by whiteflies with their respective helper begomoviruses. Betasatellites are thought to facilitate the movement of monopartite begomoviruses. They also encode a single protein (βC1) that enhances symptoms through its action as a suppressor of host gene silencing (40–42). Some alphasatellites attenuate disease symptoms (43), while others contribute to silencing suppression during infection (44).

During a CMD survey in Tanzania in 2002 and subsequent years, unusual disease symptoms, such as filiform-shaped leaves, were seen on CMB-infected cassava. This observation led to the discovery and description here of two novel DNAs, designated SEGS-1 and SEGS-2 (sequences enhancing geminivirus symptoms). Further investigations showed that sequences related to SEGS-1 and SEGS-2 occur in the cassava genome, distinguishing them from the betasatellite and alphasatellite DNAs associated with other geminivirus disease complexes.

## MATERIALS AND METHODS

### Field-infected cassava plants.

Leaf samples were collected from naturally infected cassava plants showing CMD symptoms in coastal Tanzania. Hardwood stem cuttings from the infected plants were planted in a growth chamber (28°C, 16/8-h light/dark cycle) at the Donald Danforth Plant Science Center (St. Louis, MO, USA). Symptoms on new leaves were monitored regularly, and total DNA was extracted as described previously (45).

### Amplification and cloning of SEGS-1 and SEGS-2 from infected cassava plants.

The primer pair Beta01 and Beta02 (46) was used to amplify SEGS-1 from total DNA extracts from CMD-infected cassava (Table 1 lists primer sequences). KpnI sites were introduced into both primers to facilitate cloning. SEGS-2 was amplified using the primers DNA-1/F and DNA-1/R with BamHI sites (30). For the amplification of SEGS-1, PCR was performed for 35 cycles with each consisting of 1 min at 94°C, 1 min at 55°C, and 2 min at 72°C. SEGS-2 was amplified using the same conditions except that the annealing temperature was 59°C. The largest PCR products were cloned into the pGEM-T Easy vector (Promega). Seven independent clones of SEGS-1 and SEGS-2 were sequenced using M13 forward and reverse primers. Internal primers were designed and used to obtain full-length sequences. The sequencing data were assembled using DNAStar software. The sequences were screened for putative protein-coding sequences using Gene Construction Kit 4.0, constrained by a minimum length of 99 bp and the presence of an ATG start codon.

**TABLE 1:** PCR primer sequences

Primer	Sequence^*a*^
Beta01	GGTACCACTACGCAGCAGCC
Beta02	GGTACCTACCCTCCCAGGGGTACAC
Cass Perox4F	GGTGCAGCGTGAGAAAGCAGTT
Cass Perox4R	GGCTGGGCTCATGCATTCTT
DNA-1/F	TGGGGATCCTAGGATATAAATAACACGTC
DNA-1/R	CTAGGATCCGGACAAATTACAAGCGTA
EACMVAfor3	GCCATTCCTCCATTGAAGAGC
EACMVArev6	CTGCTAACGCGGATCGAATC
SATIIF	GCCGCACCACTGGATCTC
SATIIR	CAGCAGCCAGTCAGGAAGTT
SATIIIF	AGGCCTCGTTACTAAAAGTGC
SATIIIR	ACCTGACGGCAGAAGGAAT
UG3A-2	CCGACAGTACCGCGATCGTA
UG3A-3	CGACTTGGAAAAGTCCAGCGTC
1-hp1F	TACGCAGCAGCCATCATCGACATC
1-4F	GGGTAGCCTCTAATCCTTCA
1-4R	TGAAGGATTAGAGGCTACCC
1-5F	GGTGAGTACTGCAACATAATTGC
1-2F	GCAGTTCAGCAGTTCAACTG
1-2R	CAGTTGAACTGCTGAACTGC
1-6R	GCAGCCAGTTAGGAAGTTATC
2-1F	GTGCTTGGGGTTGTATTCTTG
2-4F	GAGCCCCGTTTAAGAATTGCA
2-4R	TGCAATTCTTAAACGGGGCTC
2-5F	GACTGTTCTGTGTGCAAGTGA
2-7F	CATGCTGTCAACGCCATTGCTG
2-hp0R	ACAGATCTCAGCACATCGGAAACA
2-5R	TCACTTGCACACAGAACAGTC
2-6F	AGGCCTCGTTACTAAAAGTGC
2-6R	GCACTTTTAGTAACGAGGCCT
2-8R	CAGCAATGGCGTTGACAGCATG

a KpnI (GGTACC) and BamHI (GGATCC) restriction sites used for cloning are underlined in the primers.

### Construction of SEGS-1 and SEGS-2 dimer clones.

The pGEM-T Easy plasmid harboring SEGS-1 (pGEM-SEGS-1) was digested with KpnI, and the SEGS-1 insert was ligated into pGEM3 (Promega) linearized with KpnI. The resulting clones were screened for double insertions of the SEGS-1 fragment using EcoRI. A clone (pGEM-2SEGS-1) containing tandem dimers of SEGS-1 was identified using multiple restriction enzymes with unique sites in the SEGS-1 sequence. A dimer of SEGS-2 (pGEM-2SEGS-2) was generated using the same strategy, except that the SEGS-2 fragment from pGEM-SEGS-2 was released using BamHI and cloned into pGEM3 linearized with BamHI.

### Infection assays.

Nicotiana benthamiana and cassava plants (5-leaf stage) were inoculated by bombardment with plasmids (100 ng/plant) containing tandem dimers of SEGS-1 (pGEM-2SEGS-1) or SEGS-2 (pGEM-2SEGS-2) alone or in combination with plasmids carrying partial dimers of the DNA-A and DNA-B components of *African cassava mosaic virus* (ACMV; GenBank accession numbers AF112352 and AF112353), *East African cassava mosaic Cameroon virus* (EACMCV; AF112354 and AF112355), or *East African cassava mosaic virus*-Uganda (EACMV-UG; AF126807 and AF126806), as described previously (11). For each DNA combination, five plants were inoculated and two plants were mock inoculated. The experiments were repeated twice. The plants were grown in a greenhouse at 28°C, and disease symptoms were monitored visually starting 3 days postinoculation (dpi) and continuing for up to 60 dpi. Total DNA was extracted from N. benthamiana plants at 14 dpi and cassava plants at 21 dpi (45). The DNA (5 μg) was resolved on 1% (wt/vol) agarose gels containing TBE buffer (90 mM Tris-borate, pH 8.0, 2 mM EDTA). The DNA was transferred to Hybond-N^+^ nylon membranes (Amersham) and hybridized to radiolabeled probes specific to DNA-A from EACMCV or EACMV-UG (11). Probes were labeled with [^32^P]dATP by random priming (47).

### Analysis of SEGS-1 and SEGS-2 sequences in cassava genomic DNA.

SEGS-1 and SEGS-2 sequences were used to search the Manihot esculenta v6.1 reference genome (http://phytozome.jgi.doe.gov/pz/portal.html) using BLASTn (Blosum 62) with an E value cutoff of 1E−10 and a word length of 11. The returned cassava sequences were filtered using a length cutoff of ≥200 bp, and their positions in the cloned SEGS sequences and coincidence with cassava genes were annotated manually. The 52-bp region in SEGS-2 not found in the above-described search was used to search the cassava v6.1 genome sequence and NCBI nucleotide database using a word length of 7 and no E value cutoff.

DNA from various cassava accessions and wild Manihot spp. were surveyed by PCR for sequences related to SEGS-1 and SEGS-2. DNA from South American cassava cultivars and wild relatives was amplified using the primer pairs SAT2F/SAT2R and SAT3F/SAT3R (Table 1) and the PCR conditions described above. For characterization of African cassava cultivars, the primer pairs 1-hp1F/1-6R and 2-1F/2-5R were used (Table 1).

Total DNA from CMB-infected cassava leaves or healthy leaves collected in fields in Cameroon or Tanzania was surveyed by PCR for SEGS-1 and SEGS-2. Infected cassava leaves from Cameroon were dried and shipped to the United States, where DNA was isolated using a DNeasy plant minikit (Qiagen). Total DNA was isolated from the Tanzanian samples prior to shipment to the United States. Total DNA was also isolated from CMB-free cassava leaves propagated through tissue culture at Delaware State University. SEGS-1 and SEGS-2 were analyzed using the convergent primer pairs 1-hp1F/1-6R and 2-7F/2-hp0R, respectively. PCR was performed with 25 cycles consisting of 1 min at 94°C, 30 s at 55°C, and 1 min at 72°C. The products resulting from the convergent primer pairs (997 bp for SEGS-1 and 1,086 bp for SEGS-2) were resolved on a 1% (wt/vol) agarose gel and stained with ethidium bromide before UV light visualization.

### Analysis of SEGS-1 and SEGS-2 episomes in cassava and whiteflies.

Total DNA from CMB-infected cassava leaves, virus-free leaves, and whiteflies or DNA from virion preparations from infected cassava leaves or whiteflies were analyzed for SEGS-1 and SEGS-2 episomes. Cassava leaf samples used in the analysis are described above. DNA was isolated from individual whiteflies collected from infected cassava in Tanzanian fields and then shipped to the United States. Virion samples (48) were generated by homogenizing infected cassava leaves or pools of 20 whiteflies in 50 mM Tris, 10 mM MgSO_4_, 0.1 M NaCl, pH 7.5, followed by low-speed centrifugation. The supernatant was subjected to 0.22-μm filtration followed by DNase I digestion (2.5 U for 3 h at 37°C) prior to shipment to the United States, where virion DNA was isolated using a QIAamp MinElute virus spin kit (Qiagen).

Total and virion DNA from cassava leaves and whiteflies was amplified by rolling-circle amplification (RCA) using a templiPhi100 DNA amplification kit (GE Healthcare) according to the manufacturer's instructions. SEGS-1 was analyzed using the divergent primer pairs 1-5F/1-2R and 1-4F/1-2R and the convergent primer pair 1-4F/1-2R, while SEGS-2 was analyzed using divergent primer pairs 2-5F/2-8R and 2-4F/2-6R and the convergent primer pair 2-6F/2-4R. PCR was performed with 40 cycles for SEGS-1, consisting of 1 min at 94°C, 1 min at 55°C for the divergent primers or 47°C for the convergent primers, and 1 min at 72°C. PCR cycle conditions for SEGS-2 were similar, except the annealing temperature was 49°C. The various PCR products were resolved on a 1% (wt/vol) agarose gel and stained with ethidium bromide for UV light visualization. The PCR products for primer pairs 1-4F/1-2R (587 bp), 2-4F/2-6R (310 bp), 1-2F/1-4R (466 bp), and 2-6F/2-4R (914 bp) were gel purified using a QIAquick gel extraction kit (Qiagen) and sequenced in both orientations using primer pairs 1-4F/1-2R, 1-2F/1-4R, 2-4F/2-6R, and 2-6F/2-4R, respectively.

The RCA products were also analyzed for the presence of EACMV and EACMV-UG DNA using the primer pairs EACMVAfor3/EACMVrev6 and UG3A-2/UG3A-3, respectively. The 691-bp product for EACMV and the 796-bp product for EACMV-UG were resolved on a 1% (wt/vol) agarose gel and stained with ethidium bromide before UV light visualization. The RCA products were also tested for cassava genomic DNA contamination using the primer pair Cass Perox4-F/Cass Perox4-R.

## RESULTS

### Cloning of SEGS-1 and SEGS-2 from EAMCV-infected cassava from Tanzanian fields.

**FIG 1:**
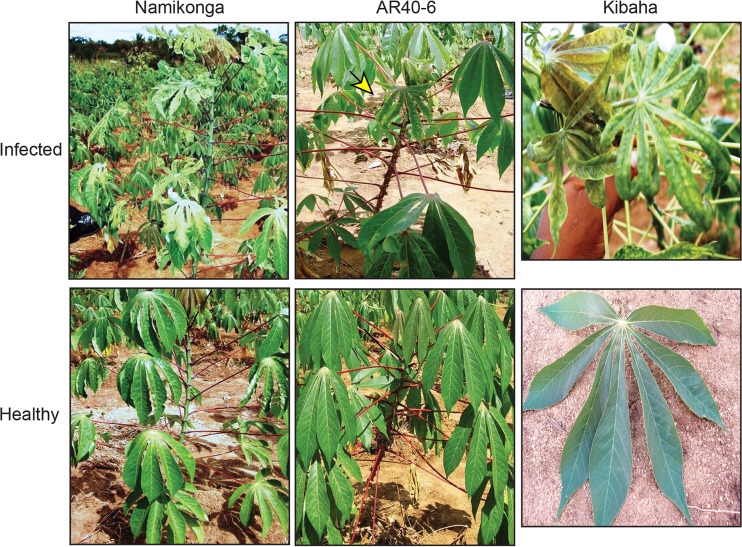
Severe symptoms in resistant cassava cultivars in Tanzanian fields. Namikonga, an Amani hybrid (Manihot esculenta × M. glaziovii), was recommended in Tanzania as CMD resistant in the 1990s. AR40-6, an International Center for Tropical Agriculture (CIAT) breeding line with the CMD2 resistance locus (4), was introduced in Tanzania in 2004. Kibaha, which is most likely an Amani hybrid, was recommended in Tanzania as CMD tolerant in the 1990s. The top panels show plants with severe symptoms associated with EACMV infection, while the bottom panels show symptom-free plants. A yellow arrow marks the symptomatic AR40-6 leaf.

Surveys of cassava fields in the coastal regions of Tanzania identified CMD symptomatic plants of resistant varieties Namikonga, AR40-6, and Kibaha (Fig. 1). The AR40-6 cultivar carries the CMD2 resistance locus. Surveys in the Tanzanian Kibaha coastal area in 2002 and later years revealed that some cassava plants displayed atypical CMD symptoms, such as leaf distortion, severe yellowing, and mosaic patterning. Other infected plants exhibited extreme leaf narrowing or filiform phenotypes. To examine this unusual breakdown in resistance, woody stem cuttings were collected, transported to the Donald Danforth Plant Science Center, and established in pots in a growth chamber. The only CMB detected in the cuttings by PCR was EAMCV, as is typical for cassava from the Kibaha region (12, 49). Hence, the unusual symptoms could not be attributed to synergism characteristic of mixed infections between EACMV and ACMV, as seen in other regions of Africa (50).

**FIG 2:**
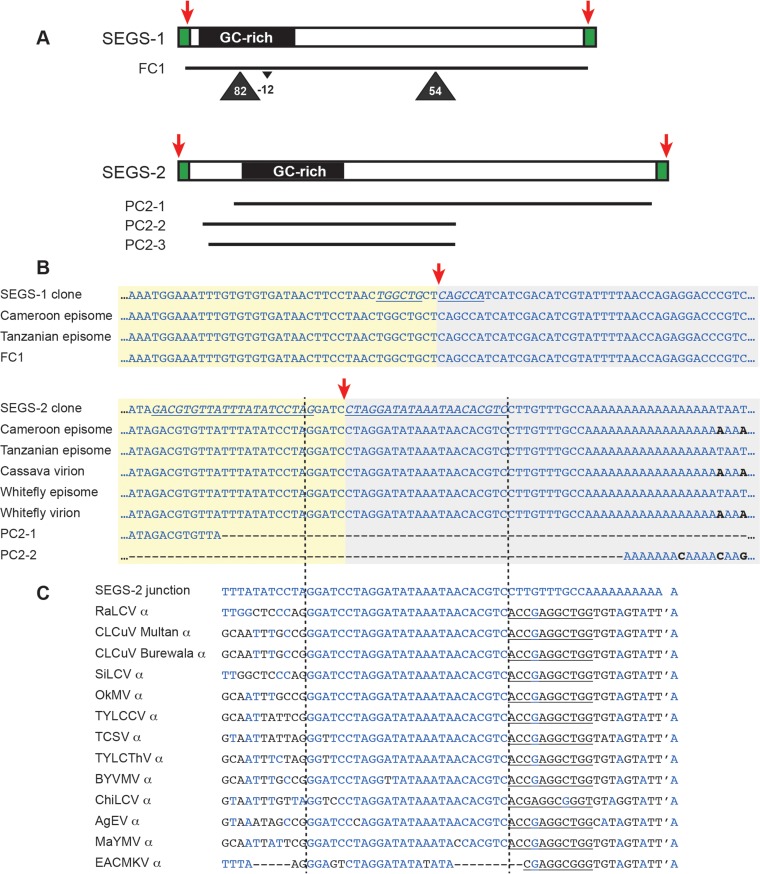
SEGS-1 and SEGS-2 clones, related cassava genomic sequences, and episomes. (A) Linear maps of SEGS-1 and SEGS-2 cloned sequences showing their GC-rich regions and flanking repeated sequences (green). The positions of cassava genomic sequences (FC1, PC2-1, PC2-2, and PC3-3; see Table S1 in the supplemental material) showing the strongest relationship to the SEGS-1 or SEGS-2 clones are marked by lines. The triangles indicate the positions and sizes of insertions or deletions detected in some genomic sequences related to SEGS-1. (B) Sequences of junction regions of SEGS-1 and SEGS-2 episomes compared to their respective clones and the longest related cassava genomic sequences. The red arrows mark the episome junctions in the linear sequences and the circular episomes (see Fig. S1 in the supplemental material for longer alignments.) The italic typeface and underlining in the clone sequences marks inverted repeats flanking the episome junctions of SEGS-1 and SEGS-2. (C) Comparison of SEGS-2 junction sequences with alphasatellite origin sequences. The vertical lines show the region of full or near identity between the two types of sequences. The apostrophe is the nick site in the alphasatellite origins, while the underlined sequences are the 5′ side of the stem structure in the origin hairpin. In panels B and C, blue typeface indicates sequence identity with the SEGS-2 clone, while black typeface marks differences. The alphasatellite sequences in panel C are associated with *Radish leaf curl virus* (RaLCV; GenBank accession number KF471057.1), *Cotton leaf curl virus* (CLCuV; HF564605.1 and HQ728354.1), *Sida leaf curl virus* (SiLCV; FR772088.1), Okra mosaic virus (OkMV; FN658718.1), *Tomato yellow leaf curl China virus* (TYLCCV; AJ888452.1), *Tobacco curly shoot virus* (TCSV; FN678903.1), *Tomato yellow leaf curl Thailand virus* (TYLCThV; FN678903.1), *Bendi yellow vein mosaic virus* (BYVMV; KF471059.1), *Chili leaf curl virus* (ChiLCV; KF471049.1), Ageratum enation virus (AgEV; FN543100.1), *Malvastrum yellow mosaic virus* (MaYMV; AM236767.1), and *East African cassava mosaic Kenya virus* (EACMKV; HE984148).

We used universal primers for betasatellites (Beta01 and Beta02 [51]) and alphasatellites (DNA1-F and DNA1-R [30]) to amplify and clone two DNAs (SEGS-1 and SEGS-2) from the CMD-infected plants displaying the severe phenotypes (Fig. 2A). Subsequent experiments showed that only one primer from each pair was necessary for amplification, e.g., Beta01 for SEGS-1 and DNA1-F for SEGS-2. Sequences related to each primer were detected at the ends of their respective products. The sequences of SEGS-1 (also called DNA-II; AY836366) and SEGS-2 (also called DNA-III; AY836367) were determined to be 1,007 and 1,197 bp in length, respectively (see Fig. S1 in the supplemental material). The differences in length between the sequences reported in Fig. S1 in the supplemental material and the NCBI entries reflect the removal of primer sequences not in the SEGS-1 or SEGS-2 genomic copies or their episomes.

SEGS-1 and SEGS-2 share only 23% sequence identity, and as such they represent distinct DNAs. Their sequences display no significant matches to any known geminivirus DNAs, indicating that they were not defective viral DNAs. Neither contains the TAATATT/AC motif that corresponds to the origin of replication in all geminiviruses and betasatellites (25, 52) or the related TAGTATT/AC motif in alphasatellites (32). Neither SEGS-1 nor SEGS-2 displays homology to betasatellite sequences, and SEGS-1 also shows no relationship to alphasatellite sequences. However, SEGS-2 contains a 26-bp sequence related to sequences located immediately upstream of the hairpin motif in several alphasatellite replication origins (Fig. 2C).

Both SEGS-1 and SEGS-2 contain regions with high GC content (SEGS-1, positions 37 to 242, 69% GC; SEGS-2, positions 151 to 379, 64% GC). The longest putative protein-coding sequences in SEGS-1 and SEGS-2 are 144 bp and 225 bp in size, respectively, and specify potential proteins that show no similarity to proteins currently in the public databases.

### SEGS-1 and SEGS-2 enhance CMD symptoms in susceptible cassava cv. 60444.

**FIG 3:**
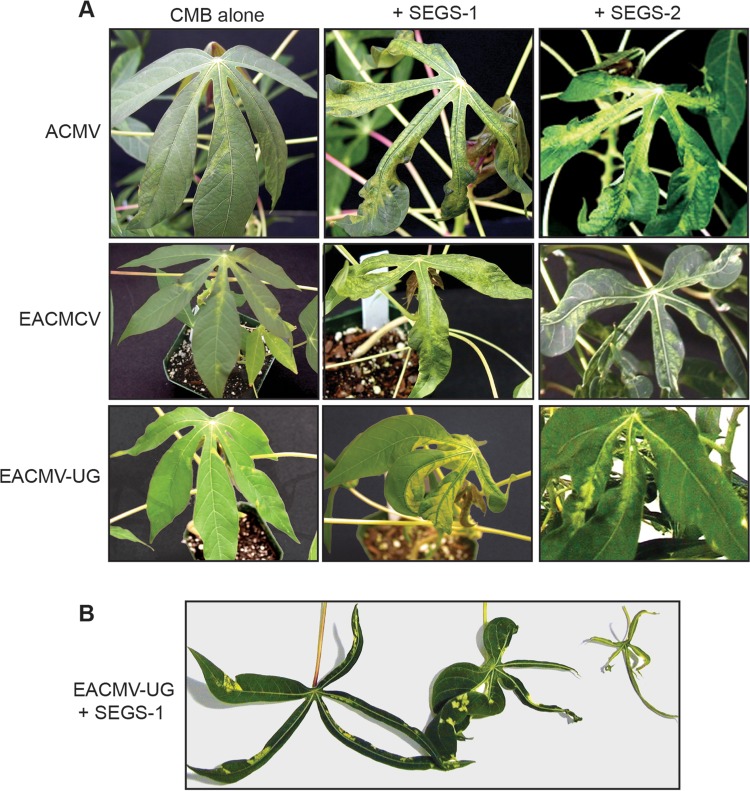
SEGS-1 and SEGS-2 enhance CMD symptoms. (A) Cassava cv. 60444 plants were bombarded with CMB DNA-A and DNA-B replicons alone or in combination with an SEGS-1 or SEGS-2 dimer plasmid under controlled conditions. (Left) Plants inoculated with ACMV, EACMCV, or EACMV-UG alone. (Middle) Each CMB coinoculated with SEGS-1. (Right) Each CMB coinoculated with SEGS-2. Photographs of representative leaves were taken at 7 dpi. (B) Range of leaf phenotypes (narrowing to extreme filiform) seen on cassava plants coinoculated with EACMV-UG and SEGS-1 at 21 dpi.

We then asked if SEGS-1 or SEGS-2 had any effects on symptoms in cassava coinoculated with three CMB species under controlled conditions. Cassava cv. 60444 plants inoculated biolistically with ACMV alone showed mild mosaic symptoms at 7 dpi (Fig. 3A). In contrast, severe mosaic symptoms developed in plants inoculated with ACMV in combination with a SEGS-1 or SEGS-2 clone at 7 dpi (Fig. 3A). Similarly, plants inoculated only with EACMCV or EACMV-UG showed mild mosaic symptoms at 7 dpi but developed severe symptoms when coinoculated with SEGS-1 or SEGS-2. In all three cases, infected cassava plants had filiform leaves, resembling the symptoms previously observed in the field, as is illustrated for leaves from plants inoculated with EACMV-UG and SEGS-1 (Fig. 3B). PCR using CMB-specific primers confirmed that all symptomatic plants contained viral DNA, including those inoculated with CMBs alone or CMBs with SEGS (not shown). The results were consistent across the 5 plants in each treatment and in two independent infection assays. Plants bombarded with SEGS-1 or SEGS-2 alone did not develop symptoms and were negative for viral DNA (not shown).

### SEGS-1 overcomes CMD2 resistance to EACMV-UG in the cassava landrace TME3.

**FIG 4:**
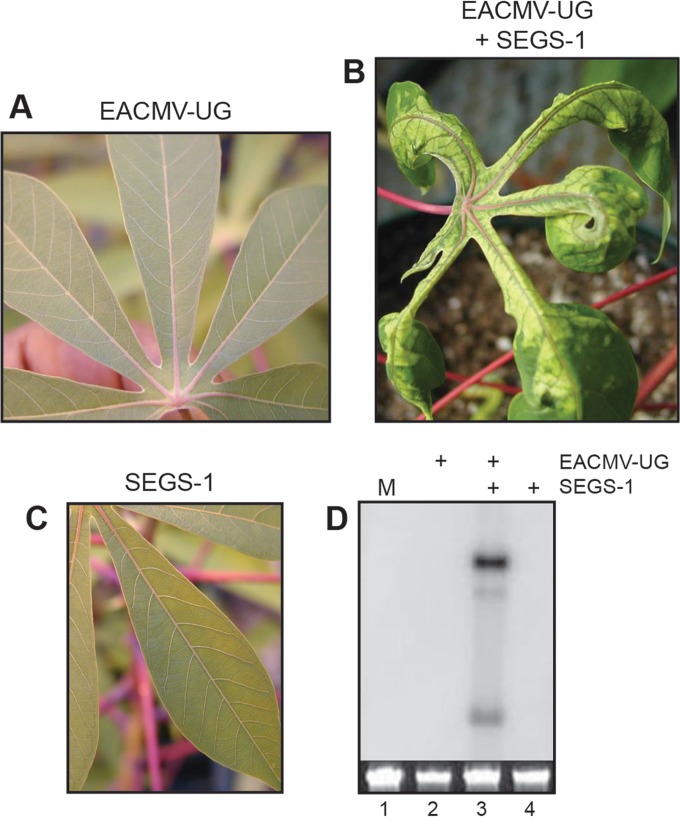
SEGS-1 can overcome CMD2 resistance of the cassava landrace TME3. TME3 plants were inoculated under controlled conditions and monitored for symptoms at 21 dpi. (A) Leaf from a plant inoculated with EACMV-UG DNA-A plus DNA-B showed no symptoms. (B) Leaf from a plant inoculated with EACMV-UG DNA-A plus DNA-B and SEGS-1 showed severe symptoms. (C) No symptoms were observed on leaves from plants inoculated with SEGS-1 alone. (D) Total DNA was extracted at 21 dpi from systemically infected leaves and equivalent leaves from symptom-free plants and was analyzed by DNA gel blotting with radiolabeled probes corresponding to EACMV-UG DNA-A. The lanes correspond to mock inoculation (M; lane 1), EACMV-UG (lane 2), EACMV-UG plus SEGS-1 (lane 3), and SEGS-1 alone (lane 4). The loading controls are ethidium bromide-stained total genomic DNA.

Like many local landraces from West Africa, TME3 carries the CMD2 resistance locus (4). CMD2 does not confer full immunity to TME3 plants; instead, it results in reduced viral titer and mild or no symptoms in the field. We found that TME3 plants are also resistant to EACMV-UG in controlled inoculation experiments, with no symptoms developing on systemic leaves at 21 dpi (Fig. 4A). Similarly, no symptoms were seen for plants bombarded with SEGS-1 alone (Fig. 4C). However, all plants coinoculated with EACMV-UG and SEGS-1 showed severe mosaic symptoms at 21 dpi (Fig. 4B), which were maintained for up to 8 months after inoculation. CMD symptoms on these plants were distinct, comprised predominantly of yellowing along leaf veins and narrowing of the leaf blade. Consistent with the symptoms, viral DNA forms were detected by DNA gel blot analysis of upper leaves from TME3 plants coinoculated with EACMV-UG and SEGS-1 at 21 dpi (Fig. 4D, lane 3) but not from plants inoculated with EACMV-UG (lane 2) or SEGS-1 alone (lane 4). TME3 plants inoculated with EACMV-UG alone or in the presence of SEGS-2 developed mild mosaic symptoms at 60 dpi, whereas plants inoculated with SEGS-2 alone were symptom free (data not shown). This result indicated that unlike SEGS-1, SEGS-2 does not alter the timing or severity of CMD symptoms in TME3.

### SEGS-2 promotes CMB infection in N. benthamiana.

**FIG 5:**
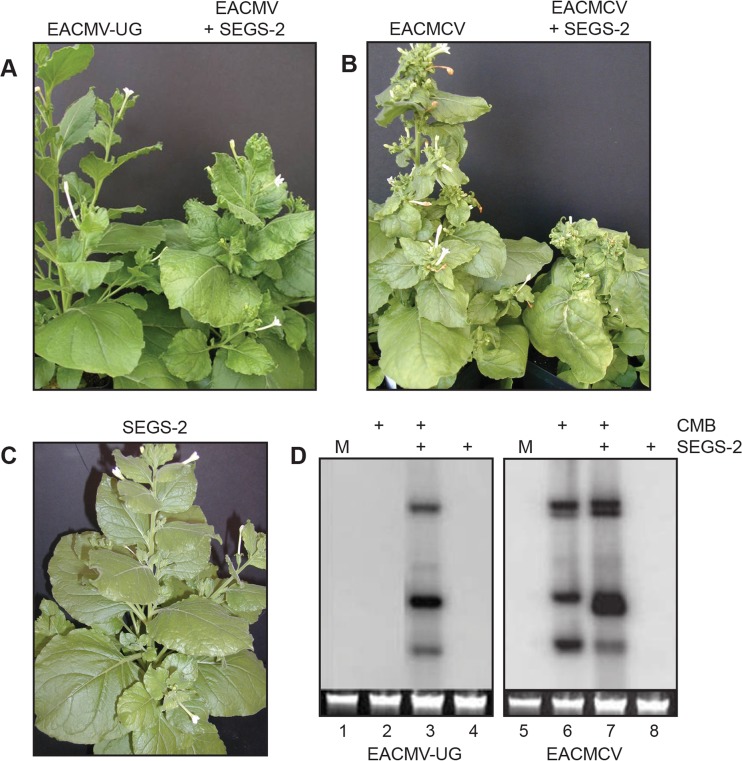
SEGS-2 enhances CMB infection in N. benthamiana. (A) N. benthamiana plants inoculated with EACMV-UG DNA-A plus DNA-B showed no symptoms, while plants coinoculated with EACMV-UG DNA-A plus DNA-B and SEGS-2 were symptomatic at 14 dpi. (B) Plants inoculated with EACMCV DNA-A plus DNA-B showed very mild symptoms, while those coinoculated with EACMCV DNA-A plus DNA-B and SEGS-2 displayed strong symptoms at 14 dpi. (C) Plants inoculated with SEGS-2 alone did not develop symptoms. (D) Total DNA was extracted at 14 dpi from systemically infected leaves and equivalent leaves from symptom-free plants and analyzed by DNA gel blotting with radiolabeled probes corresponding to EACMV-UG DNA-A (lanes 1 to 4) or EACMCV DNA-A (lanes 5 to 8). The lanes correspond to mock inoculation (M; lanes 1 and 5), CMB alone (lanes 2 and 5), CMB plus SEGS-2 (lanes 3 and 7), and SEGS-2 alone (lanes 4 and 8). The loading control is ethidium-stained, total genomic DNA.

Earlier studies reported the inability to infect N. benthamiana with EACMV-UG by biolistic inoculation (10, 11). We were also unable to infect N. benthamiana plants with EACMV-UG alone (Fig. 5A). In contrast, when EACMV-UG was coinoculated with SEGS-2, moderate systemic mosaic symptoms were observed at 7 dpi with maximum severity at 21 dpi, indicating that SEGS-2 can help EACMV-UG establish disease and express symptoms in N. benthamiana. Viral DNA was detected on DNA gel blots when EACMV-UG was inoculated with SEGS-2 (Fig. 5D, lane 3) but not when EAMCV-UG (lane 2) or SEGS-2 (lane 4) was inoculated separately. Moreover, N. benthamiana plants inoculated with EACMCV displayed mild symptoms, whereas plants coinoculated with EACMCV and SEGS-2 were severely stunted (Fig. 5B). EACMCV DNA levels showed a small increase in the presence of SEGS-2 at 14 dpi (Fig. 5D, lanes 6 and 7). The results were consistent between plants in each treatment group and between replicas of the infection assays. SEGS-1 did not result in increased symptom severity or higher viral DNA levels in N. benthamiana plants coinoculated with EACMV-UG or EACMCV, indicating that SEGS-1 does not enhance CMB symptoms in N. benthamiana.

### Cassava genomic and expressed sequence tag databases contain sequences related to SEGS-1 and SEGS-2.

We searched the recently released v6.1 annotation of the Manihot esculenta reference genome (53) for sequences related to SEGS-1 or SEGS-2 (Fig. 2A; also see Table S1 and Fig. S1 in the supplemental material). Our search uncovered a sequence (FC1) on chromosome 11 with 99% identity to full-length SEGS-1. Unlike SEGS-1, the cassava genome does not contain a sequence related to full-length SEGS-2. Instead, we found 3 partial sequences (PC2-1, 84% identity, positions 130 to 1181; PC2-2, 88% identity, positions 37 to 657; PC2-3, 89% identity, positions 56 to 657) that gave E values of 0 in the BLAST search. All three partial sequences are on chromosome 13 and together span a 45-kb region. PC2-2 and PC2-3 are adjacent to other chromosomal sequences that displayed lower identity to parts of the SEGS-2 clone. We did not find sequences matching the first 36 bp or the last 16 bp of the SEGS-2 clone in the cassava genome. The 52-bp sequence is present in all of the SEGS-2 episome junction sequences (Fig. 2B), which were amplified using primers distinct from the sequence not in the cassava genome. Hence, the absent sequence was not derived from the DNA1-F primer used to amplify the SEGS-2 clone. When the 52-bp region was queried against the NCBI nucleotide database, alphasatellite origin sequences were retrieved (E value of 0.006) but no other sequences were found (Fig. 2C).

Analysis of FC1, PC2-1, PC2-2, and PC2-3 revealed that they all include the GC-rich regions found in SEGS-1 and SEGS-2. FC1 also retains the largest potential protein-coding sequence in the SEGS-1 clone, but the longest potential protein-coding sequence in the SEGS-2 clone is not included in PC2-1, PC2-2, or PC2-3. One of the four nucleotide differences between the SEGS-1 clone and FC1 corresponds to an single-nucleotide polymorphism (SNP; 8696639) with a 69% frequency in the 57 sequenced M. esculenta strains. Of the 31 positions in the SEGS-2 clone with no matches in PC2-1, PC2-2, or PC2-3, only 5 correspond to known cassava SNPs, e.g., 89737083, 2%; 89737390, 33%; 89737396, 26%; 89737397, 5%; and 89737400, 2% (the percentage is the frequency of the SNP).

We also identified 17 partial copy sequences related to SEGS-1 (PC1) and 58 additional partial copy sequences related to SEGS-2 (PC2) in the cassava genome using the criteria of an E value of ≤−10 and a ≥200-bp match (see Table S1 in the supplemental material). The same sequences were recovered when transposable elements were masked out of the cassava genome database, indicating that they are not related to known plant transposons. The PC1 sequences are distributed over 10 of the 18 cassava chromosomes and one unmapped scaffold. The PC2 sequences are located on 16 chromosomes and one unmapped scaffold. Sequence alignment revealed that the various PC1 and PC2 sequences are different, but many have similar endpoints and include the GC-rich sequences characteristic of the SEGS clones. An interesting feature of many of the PC1 and PC2 sequences is that they consist of two tandem overlapping or closely spaced SEGS-related sequences (see Table S1 in the supplemental material for details of the repeat arrangements).

None of the genomic sequences related to SEGS-1 or SEGS-2 coincided with the 147 miRNA loci annotated in the cassava genome (54) or with the CMD2 resistance locus, which has been mapped to scaffold 05214 on cassava chromosome 12 (6, 55). Moreover, no matches for SEGS-1 and SEGS-2 were found in the genome of the closely related species Ricinus communis. Based on these observations, we concluded that the genomic sequences related to SEGS-1 or SEGS-2 represent distinct loci in the cassava genome, with many shared features.

We examined the positions of the genomic sequences related to SEGS-1 or SEGS-2 relative to transcripts listed in the v6.1 database (see Table S1 in the supplemental material). FC1 overlaps a cassava gene (Manes.11G058400) of unknown function, while PC2-1 and PC2-3 are located in genes encoding a putative RNA helicase (Manes.13G073000) and a pentatricopeptide repeat (PPR) protein (Manes.13G072800), respectively. PC2-2 is not associated with a known gene. Eight of the 17 PC1 sequences (47%) and 48 of the remaining 58 PC2 sequences (83%) also overlap or are close to known transcripts. In total, 43 genes associated with SEGS-related genomic sequences currently have functional assignments in the cassava database. Interestingly, most have been assigned functions in chromatin structure, RNA synthesis/processing, or protein synthesis/transport. The positions and orientations of the PC1 sequences relative to those of the genes are variable. In contrast, all PC2 sequences are oriented in the direction of transcription and overlap 5′ untranslated region (UTR) intron sequences or are upstream of incomplete transcripts that lack mapped 5′ UTRs.

### Sequences related to SEGS-1 and SEGS-2 are widespread in cassava and its relatives.

**FIG 6:**
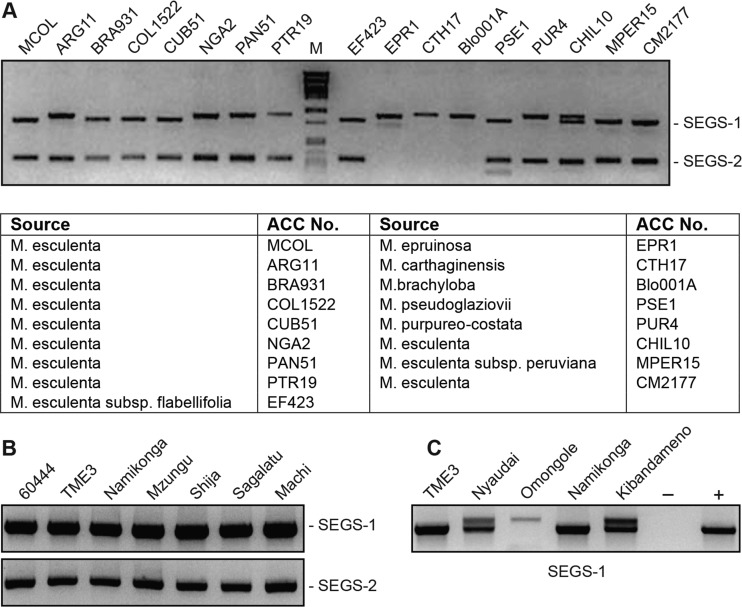
Manihot genomes contain sequences related to SEGS-1 and SEGS-2. (A) PCR analysis of genomic DNA from South American Manihot genotypes using the SATIIF/R primer pair for SEGS-1 and the SATIIIF/R primer pair for SEGS-2. The table lists the 10 South American cassava cultivars and the 7 wild Manihot species that were analyzed. (B) PCR analysis of genomic DNA from African cassava cultivars using the 1-hp1F/1-6R primer pair for SEGS-1 and the 2-1F/2-5R primer pair for SEGS-2. The DNA samples were from plants rendered virus free by passage through tissue culture. (C) Multiple PCR products related to SEGS-1 were resolved for some African cultivars.

We then asked if sequences related to SEGS-1 or SEGS-2 occur in diverse Manihot accessions. For these studies, we used primer pairs specific to SEGS-1 (SATIIF/R) or SEGS-2 (SATIIIF/R) that included one primer in the GC-rich region and one outside the GC-rich region. The SEGS-1 primers produced a fragment of about 900 bp spanning almost its entire length, while the SEGS-2 primer pair amplified a ca. 300-bp fragment (Fig. 6A), corresponding to the expected sizes of the predicted products. The primer pairs were used to amplify genomic DNA from 10 cassava strains and 7 wild Manihot spp. from South America (Fig. 6A). Products were obtained for all of the M. esculenta samples for both the SEGS-1 and SEGS-2 primer pairs. SEGS-1 products were also seen for all 7 of the wild Manihot spp., while SEGS-2 products were seen for 4 wild species.

We also used SEGS-1- and SEGS-2-specific primers to amplify genomic DNA from 7 East African cassava cultivars. In this case, the primer pairs 1-hp1F/1-6R and 2-1F/2-5R amplified about 1-kb and 600-bp fragments, respectively. Both primer pairs detected SEGS-1- and SEGS-2-related sequences in all 7 cultivars (Fig. 6B). Sequencing the PCR products confirmed that they corresponded to SEGS-1 and SEGS-2 (data not shown). The sizes of the SEGS-2 products appeared uniform, while the SEGS-1 products varied from 1 to 1.1 kb in size (Fig. 6C). Sequencing revealed that the size differences reflected the presence of one or two insertions that are conserved with respect to their sequences and positions in the different SEGS-1 PCR products (Fig. 2A, triangles). Sequences related to FC1, PC2-1, PC2-2, and PC2-3 were detected in the 57 M. esculenta accessions annotated in cassava v6.1 (Phytozome 10.3), consistent with the PCR data that the genomic copies are conserved across cassava genotypes.

### Episomal copies of SEGS-1 and SEGS-2.

**FIG 7:**
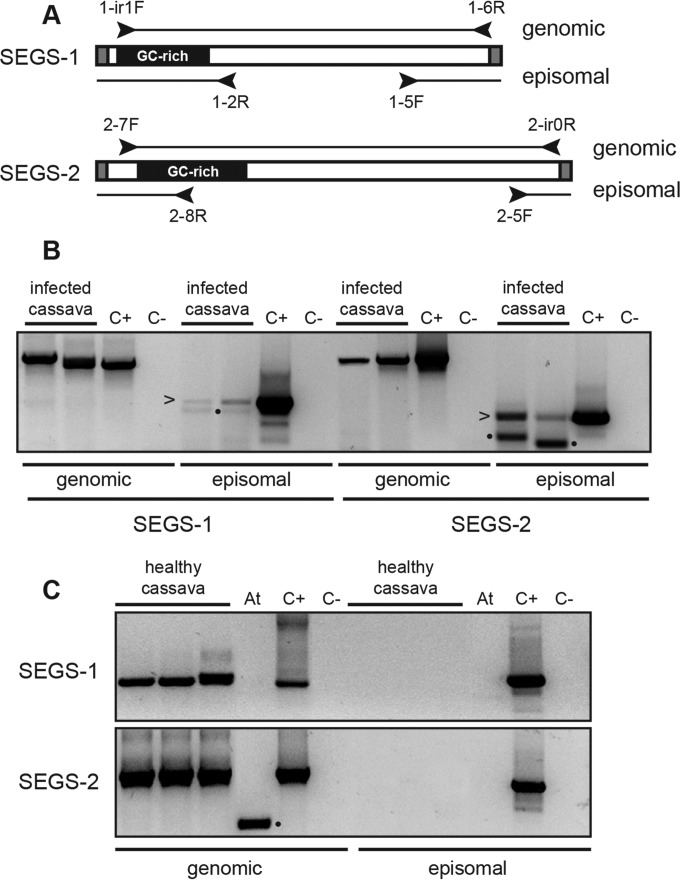
Amplification of SEGS-1 and SEGS-2 episomes in infected plants. (A) The convergent primer pairs 1-hp1F/1-6R and 2-7F/2-hp-0R amplify genomic copies of SEGS-1 and SEGS-2, respectively. The divergent primer pairs 1-2R/1-5F and 2-8R/2-5F amplify circular episomal or concatemeric copies of SEGS-1 and SEGS-2, respectively. Total DNA was the template for the genomic PCR products, while RCA DNA was the template for the episomal PCR products. (B) PCR products from CMB-infected cassava samples from Cameroon. The arrowheads mark bands with sequences that match SEGS-1 or SEGS-2. (C) PCR products from healthy cassava collected from Cameroon and passaged through tissue culture. The Arabidopsis thaliana (At) DNA was a control for potential contamination during DNA isolation. C+ is the positive PCR control using a cloned DNA template. C− is the negative PCR control that lacks template DNA. Bands marked with dots are nonspecific products that were also sequenced.

Given that SEGS-1 and SEGS-2 originally were amplified from CMD-infected cassava using primers for betasatellites and alphasatellites, we asked if they occur as episomes in cassava. We were unable to detect small DNAs corresponding to the predicted sizes of the SEGS-1 or SEGS-2 episome on DNA gel blots. Hence, we designed two types of primer pairs, a convergent set to amplify linear genomic copies and a divergent set to amplify circular episomal or concatemeric copies of SEGS-1 and SEGS-2 (Fig. 7A). The primer pairs were used for PCR of total DNA extracts from CMB-infected cassava (Fig. 7B) collected in Cameroon. We also performed PCR using DNA samples from healthy cassava plants (Fig. 7C) from Cameroon that had been passaged through tissue culture to ensure that they were virus free. The convergent primer pairs amplified the genomic sequences related to SEGS-1 or SEGS-2 from both the healthy and infected plant samples. In contrast, no PCR products were detected when the divergent primers were used to amplify the total DNA samples from healthy or infected plants. However, when the same DNA samples first were subjected to RCA to amplify small, circular DNA molecules and then amplified using the divergent primer pairs, we detected PCR products for SEGS-1 and SEGS-2 in infected plants, indicative of episomal or concatemeric forms. The episomal PCR products were detected using 40 cycles and an RCA template, while the genomic PCR products were detected with 25 cycles and a total DNA template. The negative PCR controls and the Arabidopsis DNA control, which was purified in parallel with the cassava DNA samples, did not amplify with the divergent primer pairs at 40 cycles, ruling out the possibility that the episomal products were due to contaminants.

**FIG 8:**
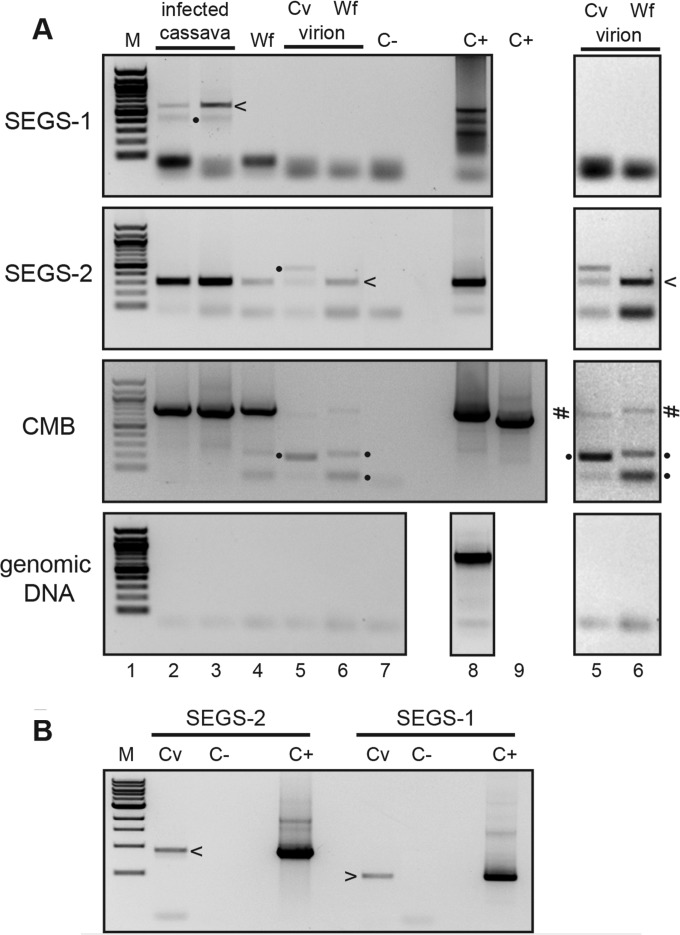
SEGS-2 episomes in infected leaves, virions, and whiteflies. (A) Divergent primer pairs were used to amplify episomal or concatameric copies of SEGS-1 and SEGS-2 from RCA template DNA. Convergent primer pairs were used to amplify CMB DNA and test for genomic DNA contamination of the RCA template DNA. The panels used the indicated primer pairs: SEGS-1 (1-4F/1-2R), SEGS-2 (2-4F/2-6R), CMB (EACMVAfor3/EACMVArev6 or UG3A-2/UG3A-3), and genomic DNA (Cass PeroxF/R). RCA template was produced using total DNA from infected cassava leaves (Cameroon, lane 2; Tanzania, lane 3), total DNA from whiteflies (Tanzania, lane 4), virion DNA from infected leaves (Tanzania, lane 5), and virion DNA from whiteflies (Tanzania, lane 6). Enhanced exposures of lanes 5 and 6 are shown at the right. C− is the negative PCR control that lacks template DNA (lane 7). C+ is the positive control using plasmid DNA corresponding to SEGS-1 (lane 8), SEGS-2 (lane 8), or CMB (lanes 8 and 9) or genomic DNA (lane 8) as the template. (B) Convergent primer pairs were used to amplify SEGS-1 or SEGS-2 in cassava leaves (Cv) from Tanzania using the same RCA template as that in lane 3 of panel A, which was shown to be free of genomic DNA. C− is the negative PCR control that lacks template DNA, while C+ is the positive control using the corresponding plasmid DNA. The arrowheads mark bands with sequences that match SEGS-2 (2-6F/2-4R) or SEGS-1 (1-2F/1-4R). Bands marked with a number sign are CMB PCR products. Bands marked with dots are nonspecific products that were also sequenced.

We then asked if SEGS-1 or SEGS-2 episomes could be detected in RCA samples generated from total DNA and virion samples from CMD-infected cassava leaves and whiteflies collected in Tanzanian fields (Fig. 8A). Both SEGS-1 and SEGS-2 episomes were detected in total DNA isolated from infected cassava leaves from Cameroon (Fig. 8A, lane 2) or Tanzania (lane 3). In contrast, SEGS-2, but not SEGS-1, episomes were detected in virion preparations from infected cassava leaves collected in Tanzania (Fig. 8A, lane 5). Similarly, only SEGS-2 episomes were detected in total DNA and virion samples from whitefly samples collected in Tanzania (Fig. 8A, lanes 4 and 6). We confirmed that the samples contained CMB DNA by convergent PCR using the primer pairs UG3A-2/UG3A-3 for EACMV-UG (Fig. 8A, lanes 2, 3, 4, and 6) and EACMVAfor3/EACMVArev6 for EACMV (lane 5). SEGS episomes were detected in a minimum of 4 independent samples of each type, with ca. half of the total DNA samples and most of the virion samples positive for episomal SEGS and CMB DNA (data not shown). SEGS-1 or SEGS-2 episomes in plants inoculated under controlled conditions (Fig. 3, 4, and 5) were not analyzed because of the presence of residual SEGS plasmid DNA, which could not be distinguished from episomal DNA in the RCA/divergent PCR assays.

Cassava genomic DNA contamination of the RCA products was ruled out using the Cassava Perox4F/R primer pair, which amplifies an 894-bp region on chromosome 16 in the cassava genome that is distinct from SEGS-related sequences (Fig. 8A, bottom). The absence of genomic DNA contamination of the RCA products allowed us to use convergent primer pairs to amplify regions of the SEGS-1 and SEGS-2 episomes not amplified by the divergent primer pairs. Bands of the same size were observed for reaction mixtures containing RCA templates of total DNA from infected cassava leaves or cloned SEGS DNA templates (Fig. 8B), indicating that the SEGS episomes and the clones are similar in size.

We characterized the junctions and the internal regions of the SEGS episomes by sequencing the divergent and convergent PCR products (Fig. 2B) amplified from the RCA templates. The junction sequences of the SEGS-1 episomes from infected cassava leaves collected in Cameroon and Tanzania were identical and joined 5′ and 3′ sequences in the SEGS-1 clone and in the genomic sequence (FC1) (red arrows in Fig. 2A and B; also see Fig. S1 in the supplemental material). The junction sequences of the SEGS-2 episomes from infected cassava leaves collected in Cameroon and Tanzania and from virion and whitefly samples collected in Tanzania were also identical except for limited variation in a downstream poly(A) tract (Fig. 2B). The SEGS-1 junction is flanked by a 6-bp inverted repeat separated by 2 bp, while the SEGS-2 junction is flanked by a 21-bp inverted repeat separated by 4 bp. The SEGS-2 episomes included an invariant 52-bp junction region that matches the 5′ and 3′ ends of the SEGS-2 clone but is absent from the cassava genome. This region includes the 26-bp sequence that is identical to sequences located immediately upstream of the hairpin motif in several alphasatellite replication origins (Fig. 2C). The internal sequences of the SEGS-1 and SEGS-2 episomes are nearly identical to their corresponding cloned sequences (see Fig. S1).

## DISCUSSION

Geminivirus infection is associated with a variety of symptoms, including leaf deformation, mosaic patterning, and stunting. The symptoms reflect virus-plant interactions that recruit and redirect host processes for viral propagation and activate defense responses (26). Some virus-host combinations result in mild symptoms, while others show severe symptoms. Symptom severity has been correlated with silencing suppressors encoded by geminiviruses and their satellites (39, 56). In this study, we report the cloning and characterization of two DNA sequences from CMB-infected cassava that enhance geminivirus disease symptoms and are designated SEGS-1 and SEGS-2. Cassava plants coinoculated with a CMB and SEGS-1 or SEGS-2 develop filiform leaf and bleaching symptoms that resemble atypical CMD symptoms observed in susceptible and resistant cassava cultivars in the field.

Geminivirus satellite DNAs often are associated with increased symptom severity (39), and initially it was thought that SEGS-1 and SEGS-2 were satellites that alter CMB symptoms. The fact that both sequences first were amplified using universal primers for geminivirus satellites contributed to this view. However, SEGS-1 and SEGS-2 show little resemblance to known geminivirus satellites. Moreover, SEGS episomes were detected only after 40 PCR cycles of RCA template DNA (Fig. 7B and 8), indicating that they are low in copy number. Together, these observations raised questions as to the origins of SEGS-1 and SEGS-2 and whether they are transmitted with CMBs.

SEGS-1, which was amplified using a betasatellite universal primer, shows no sequence relationship to betasatellites except for 16 and 9 nucleotides of the Beta01 primer at its 5′ and 3′ ends, respectively. The cassava genome contains a sequence that is 99% identical to full-length SEGS-1. In contrast, the cassava genome does not contain a sequence corresponding to full-length SEGS-2, which was amplified using an alphasatellite primer. SEGS-2 shows only 84 to 89% identity to 3 cassava genomic sequences that together encompass 1,145 bp of the 1,197-bp SEGS-2 clone. Comparison of the average number of SNPs across 57 cassava accessions in the Phytozome database uncovered fewer SNPs in PC2-1, PC2-2, and PC2-3 (27.5/1,000 nt) than in FC1 (80/1,000 nt). Thus, it is unlikely that natural variation contributed to the lower sequence identity of the SEGS-2 clone relative to those of the related genomic sequences. Moreover, sequences related to SEGS-1 occur in all 7 wild cassava relatives from South America, while sequences related to SEGS-2 were detected only in some wild relatives (Fig. 6A). Based on these observations, we think that the SEGS-1 clone, but not the SEGS-2 clone, was amplified from a cassava genomic DNA template.

We used a combination of RCA and divergent PCR to amplify and characterize episomes corresponding to the SEGS (Fig. 7 and 8). SEGS-1 and SEGS-2 episomes were detected in infected, but not healthy, cassava leaves collected in Cameroon and Tanzania, where severe CMD symptoms have been observed in cassava fields. We also detected SEGS-2 episomes in whiteflies and virions prepared from infected leaves and whiteflies collected in Tanzania, but we were unable to detect SEGS-1 episomes in the same samples. The absence of SEGS-1 indicates that the detection of the SEGS-2 episome is not due to the contamination of the virion preparations by unpackaged SEGS DNA. These results strongly suggest that SEGS-2 episomes are packaged into virions and transmitted by whiteflies along with CMBs.

Strikingly, the sequences of all of the SEGS-2 episomes and our SEGS-2 clone are nearly identical internally and across the junction region, including the 52-bp sequence that does not occur in the cassava genome and contains a 26-bp motif related to alphasatellite origins (Fig. 2). This strong level of sequence conservation, which is maintained in the SEGS-2 episomes from infected leaves collected in both Cameroon and Tanzania, suggests that this region plays an essential role in SEGS-2 function and/or propagation. One possible scenario is that the template molecule for SEGS-2 amplification arose through a recombination event between an alphasatellite and cassava genomic DNA in the past. The reduced level of identity of the SEGS-2 clone to related sequences in the cassava genome might reflect rolling-circle replication of the SEGS-2 episome during infection and the accumulation of mutations over time, as has been observed for begomoviruses (13). Recently, an alphasatellite was identified in geminivirus-infected cassava in Madagascar (36), but there are no reports of alphasatellites associated with CMD on the African continent.

Our failure to detect SEGS-1 episomes in whitefly and virion samples lends support to the hypothesis that it is derived from the cassava genome. Rolling-circle amplification of total DNA from mouse and human cells detected many extrachromosomal, closed circular DNAs that are related to nonrepetitive genomic sequences and are thought to be by-products of chromosomal DNA replication (57). The SEGS-1 episome has many features in common with these extrachromosmal DNAs, including its low copy number, lack of relationship to known repetitive elements, and presence of small direct repeats flanking the ends of the full copy in the cassava genome. Moreover, the SEGS-1 episome has been detected only in infected plants that have been reprogrammed to support both viral and plant DNA replication (58, 59), raising the possibility that the SEGS-1 episome is a by-product of host DNA replication induced by geminivirus infection (58, 60). In support of this idea, the direct repeats (GCTGCA) at the ends of genomic sequence related to SEGS-1 coincide with the junction sequence of the SEGS-1 episome. A sequence related to SEGS-1 (98% identity) was cloned using betasatellite primers from begomovirus-infected Mentha plants showing severe leaf deformation (61), suggesting that an SEGS-1 episome underwent lateral transfer, possibly in association with geminivirus infection. However, the failure to detect SEGS-1 episomes in virions is not consistent with this possibility, and the source of SEGS-1 in Mentha remains elusive.

SEGS-1 and SEGS-2 interact with CMBs differently depending on the viral and plant species. Both SEGS enhance ACMV, EACMCV, and EACMV-UG symptoms in susceptible cassava cv. 60444. SEGS-1 (but not SEGS-2) is also associated with enhanced disease symptoms and increased viral DNA accumulation in the TME3 landrace coinoculated with EACMV-UG. In contrast, SEGS-2 (but not SEGS-1) promotes EACMV-UG infection and enhances EACMCV symptoms in N. benthamiana. These differences suggest that SEGS-1 and SEGS-2 target distinct processes involved in geminivirus infection or host defense. However, the enhancement of CMB infection by SEGS-2 in N. benthamiana and the presence of an SEGS-1-related sequence in infected Mentha plants showing severe leaf deformation (61) suggest that both have the potential to alter infection in diverse plant species.

The sequences of SEGS-1 and SEGS-2 provide few clues as to the nature of their products and how they might function. The longest coding regions in SEGS-1 and SEGS-2 specify proteins of 52 and 75 amino acids in length, and neither shows significant homology to known proteins or domains. FC1 maps to the center of a gene of unknown function, while PC2-2 and PC2-3 are located in the 5′ UTRs of genes encoding a putative RNA helicase and a PPR protein, respectively. Strikingly, all 50 of the SEGS-2 partial copy sequences associated with genes map to the 5′ end or upstream of gene annotations that are truncated at the 5′ end. Moreover, the SEGS-2 partial copy sequences that overlap 5′ UTRs contain a conserved splice donor site. If the SEGS-2 episome is transcribed, its RNA might bind to splicing factors necessary to process cassava transcripts that contain related splice sites in their 5′ UTRs. Given that many of these transcripts specify proteins involved in chromatin structure, RNA synthesis/processing, or protein synthesis/transport, altering their splicing and potentially their translatability could impact host factors that influence geminivirus infection. As an example, geminivirus genomes assemble into minichromosomes (62), and changes in the host machinery that modulate chromatin could alter viral replication and/or transcription (63). A BLAST search did not detect SEGS-related sequences in the N. benthamiana genome, indicating that the ability of SEGS-2 to promote CMB infection is not dependent on the presence of related sequences in the host genome.

A key question is whether the genomic sequences related to SEGS-1 or SEGS-2 can enhance CMD symptoms or break resistance. All cassava accessions tested to date contain the genomic SEGS sequences, including cv. 60444 and TME3, which were used for the experiments showing that exogenous SEGS DNA coinoculated with CMBs enhances CMD symptoms and overcomes resistance. These results suggested that the genomic SEGS copies either were not active or not maximally active in the infection experiments. The cloned SEGS-2 sequence differs significantly from the related genomic sequences, raising the possibility that the genomic sequences cannot support symptom enhancement. In contrast, the cloned SEGS-1 sequence and its corresponding genomic sequence are 99% identical and are predicted to have the same potential to impact CMD. Sequencing the endogenous SEGS-1 sequences in cv. 60444 and TME3 did not uncover any SNPs (not shown), ruling out cultivar differences that might explain why SEGS-1 genomic sequences did not impact CMD symptoms in the controlled inoculation experiments. One possibility is that the chromatin context of the SEGS-1 genomic sequence suppresses its activity, while exogenous SEGS-1 DNA is not subject to this suppression. Recent studies have highlighted the role of epigenetic regulation on plant defense genes (64–66) and implicated environmental factors in epigenetic regulation and defense (67, 68), and the role of epigenetics currently is being assessed in the functionality of CMD2 resistance (Nigel Taylor, personal communication). Thus, if the SEGS-1 genomic sequence is controlled epigenetically, environmental or other external factors might modulate its activity and/or the release of an active SEGS-1 episome from the cassava genome, thereby providing a potential mechanism for the appearance of the atypical, severe CMD symptoms seen in resistant cultivars in African fields in recent years.

The CMD pandemic in sub-Saharan African countries has been attributed primarily to synergism and genetic recombination between EACMV, EACMV-UG, and ACMV (69). SEGS-1 and SEGS-2 also enhance CMD, resulting in atypical symptoms characterized by extreme leaf deformation, and SEGS-1 can overcome CMD2-mediated resistance in controlled inoculation experiments. Similar CMD symptoms have been reported in fields across Africa, often planted with resistant cassava cultivars, some of which carry the CMD2 resistance locus. Thus, a better understanding of the origins of SEGS-1 and SEGS-2, the distributions of their episomes, and their capacities to enhance CMD symptoms and break CMD2 resistance under suitable field conditions is essential for the development of effective and sustainable disease control measures against geminivirus diseases in cassava.

## Supplementary Material

Supplemental material
