# Supplementary material for: Two Novel DNAs That Enhance Symptoms and Overcome CMD2 Resistance to Cassava Mosaic Disease
Source: J Virol. 2016 Mar 28;90(8):4160–73. doi: 10.1128/JVI.02834-15 (PMC4810563; doi:10.1128/JVI.02834-15)
Supplement: Supplemental material [file supp_90_8_4160__index.html]

Two Novel DNAs That Enhance Symptoms and Overcome CMD2 Resistance to Cassava Mosaic Disease — Supplemental material 

# Two Novel DNAs That Enhance Symptoms and Overcome CMD2 Resistance to Cassava Mosaic Disease

## Supplemental material

- Supplemental file 1 -

  Fig. S1 (Alignment of cloned SEGS sequences with their corresponding full-copy and partial-copy sequences in the cassava reference genome and with the junction regions of their episomes.)

  Table S1 (SEGS sequences in the cassava reference genome.)

  PDF, 181K
