# Supplementary material for: Two Novel DNAs That Enhance Symptoms and Overcome CMD2 Resistance to Cassava Mosaic Disease
Source: J Virol. 2016 Mar 28;90(8):4160–73. doi: 10.1128/JVI.02834-15 (PMC4810563; doi:10.1128/JVI.02834-15)
Supplement: Supplemental material [file JVI.02834-15_zjv999091566so1.pdf]

Supplementary Figure 1 - Alignment of cloned SEGS sequences with their corresponding full copy and partial copy sequences in the cassava reference genome (v6.1) and with the junction regions of their episomes. The GC-rich regions are underlined. Sequences differences are indicated in black. The 52-bp region in SEGS-2 that is not in the cassava reference genome is shown in red, with the sequence related to alphasatellite origins highlighted by yellow. Sequences outside the junction regions that were amplified using convergent primers are indicated in green uppercase type. The primer sequences and the corresponding sequences derived from the primers in the SEGS clones are in lowercase green type. Positions with dashes indicate the absence of the sequence while blank regions indicate that no sequence information is available.

SEGS-1

```
                                   ggtaccactacgctacgcagcagcc
SEGS-1                              gtaccactacgctacGCAGCAGCCATCATCGACATCGTATTTTAACCAGAGGACCCGTCG
Cameroon Episome                    -------------------CAGCCATCATCGACATCGTATTTTAACCAGAGGACCCGTCG
Tanzania Episome                    -------------------CAGCCATCATCGACATCGTATTTTAACCAGAGGACCCGTCG
Cassava Genome                      ---------ACGCT--GCAGCAGCCATCATCGACATCGTATTTTAACCAGAGGACCCGTCG

SEGS-1                              ACCGCCTGAGCAGCAGCACGTCGCACCAGCACCACCGCCGCATCGCGCGCCTGTGAGCCG
Cameroon Episome                    ACCGCCTGAGCAGCAGCACGTCGCACCAGCACCACCGCCGCATCGCGCGCCTGTGAGCCG
Tanzania Episome                    ACCGCCTGAGCAGCAGCACGTCGCACCAGCACCACCGCCGCATCGCGCGCCTGTGAGCCG
Cassava Genome                      ACCGCCTGAGCAGCAGCACGTCGCACCAGCACCACCGCCGCATCGCGCGCCTGTGAGCCG

SEGS-1                              CCGCATCACTGGATCTCGTGCTCGTGAGCCGCCGCACGCCGCAACTCTTCATCTACCGCT
Cameroon Episome                    CCGCATCACTGGATCTCGTGCTCGTGAGCCGCCGCACGCCGCAACTCTTCATCTACCGCT
Tanzania Episome                    CCGCATCACTGGATCTCGTGCTCGTGAGCCGCCGCACGCCGCAACTCTTCATCTACCGCT
Cassava Genome                      CCCCACCACTGGATCTCGTGCTCGTGAGCCGCCGCACGCCGCAACTCTTCATCTACCGCT

SEGS-1                              CGTTTACAGCCCACCTCCGTATCACGCGATTGTGAGCCGCCGACTGCCCGCCGCACGCCC
Cameroon Episome                    CGTTTACAGCCCACCTC-GTATCACGCGATTGTGAGCCGCCGACTGCCCGCCGCACGCCC
Cassava Genome                      CGTTTACAGCCCACCTCTGTATCACGCGATTGTGAGCCGCCGACTGCCCGCCGCACGCCC

SEGS-1                              GCACCTCTGCATCAACTGCTCGTTTGCCACCCACCTCGCTCCTCTGCAGTTCAGCAGTTC
Cameroon Episome                    GCACCTCTGAATCAACTGCTCGTTTGCCACCCACCTCGCTCCTCTGCAGTTCAGCAGTTC
Tanzania Episome                                                                TGCAGTTCAGCAGTTC
Cassava Genome                      GCACCTCTGCATCAACTGCTCGTTTGCCACCCACCTCGCTCCTCTGCAGTTCAGCAGTTC

SEGS-1                              AACTGTAAGCATTTTTTCGTTAAATCTGAAGAAAATAGTTCTGGATAGAATTTTGATTGG
Cameroon Episome                    AACTG
Tanzania Episome                    AACTGTAAGCATTTTTTCGTTAAATCTGAAGAAAATAGTTCTGGATAGAATTTTGATTGG
Cassava Genome                      AACTGTAAGCATTTTTTCGTTAAATCTGAAGAAAATAGTTCTGGATAGAATTTTGATTGG

SEGS-1                              TAAGCATTATGAATTTATTATGACATTCAAGTTTATAGGCATCATAGTGTTGCTTAGACA
Tanzania Episome                    TAAGCATTATGAATTTATTATGACATTCAAGTTTATAGGCATCATAGTGTTGCTTAGACA
Cassava Genome                      TAAGCATTATGAATTTATTATGACATTCAAGTTTATAGGCATCATAGTGTTGCGTAGACA

SEGS-1                              TACTTAGCTTGTAGTTCCAGAAAATAGAGTCATTTCTGGTTTTCTTTTACAATGGAGGTG
Tanzania Episome                    TAC-TAGCTTGTAGTTCCAGAAAATAGAGTCATTTCTGGTTTGCTTTTACAATGGAGGTG
Cassava Genome                      TACTTAGCTTGTAGTTCCAGAAAATAGAGTCATTTCTGGTTTTCTTTTACAATGGAGGTG

SEGS-1                              TTTATTCCATTGTAATTTTGAGCTGAGCTTTGTTAAGGACCTTTGGAGCTCGAGCTTTGT
Tanzania Episome                    TTTATTCCATTGTAATTTTGAGCTGAGCTTGGTTAAGGACCTTTGGAGCTCGAGCTTTGT
Cassava Genome                      TTTATTCCATTGTAATTTTGAGCTGAGCTTTGTTAAGGACCTTTGGAGCTCGAGCTTTGT

SEGS-1                              TTACAAGCATCTTGATAGAGCTTTTCGAGCTCGAATTAGAATTAGGCTCATGGTTATACT
Tanzania Episome                    TTACAAGCATCTTGATAGAGCTTTTCGAGCTCGAATTAGAATTAGGCTCATGGTTATACT
Cassava Genome                      TTACAAGCATCTTGATAGAGCTTTTCGAGCTCGAATTAGAATTAGGCTCATGGTTATACT

SEGS-1                              AAAGGAGTTTTTCATGAGTTTGAGTGCTTCCAAAATTTTTTAATAAAAGCTTTACAAAGC
Tanzania Episome                    AAAGGAGTTTTTCATGAGTTTGAGTGCTTCCAAAATTTTTTAATAAAAGCTTTACAAAGC
Cassava Genome                      AAAGGAGTTTTTCATGAGTTTGAGTGCTTCCAAAATTTTTTAATAAAAGCTTTACAAAGC
```

```
SEGS-1                  TCAGCTTGGATCGATTACACCTCTACTGACCCTACTCAGTTTGGGACTCTGGCTGGGGCC
Tanzania Episome        TCAGCTTGGATCGATTACACCTCTACTGACCCTACTCAGTTTGGGACTCTGGCTGGGGCC
Cassava Genome          TCAGCTTGGATCGATTACACCTCTACTGACCCTACTCAGTTTGGGACTCTGGCTGGGGCC

SEGS-1                  ATTCTCAAAAGCCATTTATCTGGGTAGCCTCTAATCCTTCAACTCTATTTTTCCGTTTGG
Cameroon Episome                                                             CGTTT-G
Tanzania Episome        ATCCTCAAAAGCCATTTATCTGGGTAGCCTCTAATCCTTCAACTCTATTTTTCCGTTTGG
Cassava Genome          ATTCTCAAAAGCCATTTATCTGGGTAGCCTCTAATCCTTCAACTCTATTTTTCCGTTTGG

SEGS-1                  TTCTGAGAGAGTACTAAAAAGGAAATCCAACCATATATGATCAAATCTAATGATATAGCT
Cameroon Episome        CTCTGAGAGAGTACTAAAAAGGAA-TCCAACCATATATGATCAAATCTAATGATATAGCT
Tanzania Episome        TTCTGAGAGAATACTAAAAAGGAAATCCAACCATATATGATCAAATCTAATGATATAGCT
Cassava Genome          TTCTGAGAGAGTACTAAAAAGGAAATCCAACCATATATGATCAAATCTAATGATATAGCT

SEGS-1                  GGTGAGTACTGCAACATAATTGCAATTTATGCAGTTATTTCTCTTGAATTTGGTATCTGC
Cameroon Episome        GGTGAGTACTGCAACATAATTGCAATTTATGCAGTTATTTCTCTTGAATTTGGTATCTGC
Tanzania Episome        GGTGAGTACTGCAACATAATTGCAATTTATGCAGTTATTTCTCTTGAATTTGGTATCTGC
Cassava Genome          GGTGAGTACTGCAACATAATTGCAATTTATGCAGTTATTTCTCTTGAATTTGGTATCTGC

SEGS-1                  AATTTATGTATAAATCCCTAGCAGAATATTTTACTGGAGTGGTGAATATGTGTAGGCTTC
Cameroon Episome        AATTTATGTATAAATCCCTAGCAGAATATTTTACTGGAGTGGTGAATATGTGTAGGCTTC
Tanzania Episome        AATTTATGTATAAATCCCTAGCAGAATATTTTACTGGAGTGGTGAATATGTGTAGGCTTC
Cassava Genome          AATTTATGTATAAATCCCTAGCAGAATATTTTACTGGAGTGGTGAATATGTGTAGGCTTC

SEGS-1                  ACTATAGTGGAAATGGAAATTTGTGTGTGATAACTTCCTAACTGGCTGCTGCgtagcgta
Cameroon Episome        ACTATAGTGGAAATGGAAATTTGTGTGTGATAACTTCCTAACTGGCTGCTGC--------
Tanzania Episome        ACTATAGTGGAAATGGAAATTTGTGTGTGATAACTTCCTAACTGGCTGCT----------
Cassava Genome          ACTATGGTGGAAATGGAAATTTGTGTGTGATAACTTCCTAACTGGCTGCTGC--------
                                                                   ggctgctgcgtagcgta
SEGS-1                  gtggtac
Cameroon Episome        -------
Tanzania Episome        -------
Cassava Genome          -------
                        gtggtacc
```

SEGS-2

```
                        cctaggatataaataacacgtc
SEGS2_Clone             CTAGGATATAAATAACACGTCCTTGTTTGCCAAAAAAAAAAAAAAAAATAATAATAATCT
Plant Virion            CTAGGATATAAATAACACGTCCTTGTTTGCCAAAAAAAAAAAAAAAAAAAAAAAAAATGC
Whitefly Virion         CTAGGATATAAATAACACGTCCTTGTTTGCCAAAAAAAAAAAAAAAAAAAAAATAATCT
Whitefly_Episome        CTAGGATATAAATAACACGTCCTTGTTTGCCAAAAAAAAAAAAAAAAATAATAATAATGT
Cameroon_Episome        CTAGGATATAAATAACACGTCCTTGTTTGCCAAAAAAAAAAAAAAAAAAAAAAAAAATTTT
Tanzania_Episome        CTAGGATATAAATAACACGTCCTTGTTTGCCAAAAAAAAAAAAAAAAATAATAATAATGT
PC2-1                   ------------------------------------------------------------
PC2-2                   ------------------------------------AAAAAAACAAAACAAGAAAAATCT
PC2-3                   -------------------------------------------------------AATCT


SEGS2_Clone             AGGCC-TCGTTACTAAAAGTGCAAAAACCAAATAACTAAACCCTCA-CTCTCCATCCCTA
Plant Virion            AGGC
Whitefly_Episome        AGGCC-TCGTTACTAAAAGTGCAAAAACCAAATAACTAAACCCTCA-CTCTCCATCCCTA
Cameroon_Episome        AGGCC-TCGTTACTAAAAGTGCAAAAACCAAATAACTAAACCCTCA-CTCTCCATCCCTA
Tanzania_Episome        AGGCC-TCGTTACTAAAAGTGCAAAAACCAAATAACTAAACCCTCA-CTCTCCATCCCTA
PC2-1                   ------------------------------------------------------------
PC2-2                   AGGCCCTCGTTACTAAAAGCGCAAAACCTAAATAACTAAACCCTCAACTCTCCATGCCTA
PC2-3                   AGGCCCTCGTTACTAAAAGCGCAAAAACTAAATAACTAAACCCTCAACTCTCCATGCCTA


SEGS2_Clone             ACATCTCGTATACTCTCAACGCAGCTGCCCGTTCCCTCCC--CCGCCCGTGTCTACCTAT
Whitefly_Episome        ACATCTCGTATACTCTCAACGCAGCTGCCCGTTCCCTCCC--CCGCCCGTGTCTACCTA-
Cameroon_Episome        ACATCTCGTATACTCTCAACGCAGCTGCCCGTTCCCTCCC--CCGCCCGTGTCTACCTAT
Tanzania_Episome        ACATCTCGTATACTCTCAACGCAGCTGCCCGTTCCCTCCC--CCGCCCCT-TCGGTCTCT
PC2-1                   -----------ACTCTCAACACTGCTGCTCGTCCCCTCCCGTCCCCCCGTGTCTACCTCT
PC2-2                   ACATCTCTTAAACTCTCAACGCAGCTGCCCGTTCCCTCCC--CCGCCCGTGTCTACCTCT
PC2-3                   ACATCTCTTAAACTCTCAACGCAGCTGCCCGTTCCCTCCC--CCGCCCGTTTCTACCTCT

SEGS2_Clone             CCGCCTCACCCTCTGGTGTAGACGTCCGCCTTCCGCCGATTGTCCCTC-----TGCTCTT
Cameroon_Episome        CCGCCTCGCCCTCGGGTGTAGACGTCCGCCTTCCGCCGATTGTCCCTC-----TGCTCTT
Tanzania_Episome        CCGCCTCACCCTCTGGTGTAGACGTCCGCCT-CCGCCGAT-GTCCCTC-----TGCTCTT
PC2-1                   CCGCCTCTCC-TCTGGTACAGCCGCGCGCCT-CCGCCTCTTGCCTATCGTCCTTGTTCTT
PC2-2                   CC-CCTCACCCTCTGGTGTAGACGTCCACCTCCCGCCGATTGTCTCTC-----TGCTGTT
PC2-3                   CCGCCTCACCCTCTGGTGTAGACGTCCGCCTCCCGCCGATTGTCCCTC-----TGCTCTT

SEGS2_Clone             CATGCTGTCAACGCCATTGCTGCATCCGGTGCTCGTTGCTGCGTCCGCTAGTCCTGGTTG
Cameroon_Episome        CATGCTGTCAACGCCATTGCTGCATCCGGTGCTCGTTGCTGCGTCCGCTAGTCCTGGTTG
Tanzania_Episome        CATGCTGTCAACGCCATTGCTGCATCCGGTGCTCGTTGCTGCGTCCGCTAGTCCTGGTTG
PC2-1                   CACGCTGTCAACG-------------------TCGTTGCTGCGTCCGCTGTTTCTGGTTG
PC2-2                   CATGCTGTCAACGGCATTGCTGCATCCGGTGCTCGTTGCTGCGTCCGCTAGTTCTGGTTG
PC2-3                   CATGCTGTCAACGCCATTGCTGCATCCGGTGCTCGTTGCTGCGTCCGCTAGTCCTGGTTG

SEGS2_Clone             CTTCTTTTCTCTCCTCCGCCGCTCCCTCTGGTCCTCGTCGTTGCATCCCCTGCTCCATTC
Cameroon_Episome        CTTCTTTTCTCTCCTCCGCCGCTCCCTCTGGTCCTCGTCGTTGCATCCCCTGCTCCATTC
Tanzania_Episome        CTTCTTTTCTCTCCTCCGCCGCTCCCTCTGGTCCTCGTCGTTGCATCCCCTGCTCCATTC
PC2-1                   CTTCATCGCTTTCCTCCGCAGCTTCCTCTGGTCCTCGTCGCTGCGTCCACTGGTCCAGTC
PC2-2                   CTTCTTTTCTCTCCTCCGCCGCTCCCTCTGGTTCTCTTCGTTGCATCCCCTGGTCCATTC
PC2-3                   CTTCTTTTCTCTCCTCCGCCGCTCCCTCTGGTCCTCGTCGTTGCATCCCCTGCTCCATTC

SEGS2_Clone             CTTCTGCCGCCCGGTGCTGCTTGTCGCCTTTGGTCCTCG-----TCCTCAATCGCACCGC
Cameroon_Episome        CTTCTGCCGCCCGGTGCTGCTTGTCGCCTTTGGTCCTCG-----TCCTCAATCGCACCGC
Tanzania_Episome        CTTCTGCCGCCCGGTGCTGCTTGTCGCCTTTGGTCCTCG-----TCCTCAATCGCACCGC
PC2-1                   ATCCTGCCGTCCGGTGCTGCTCCTTGCCGCTGGTCCTCGCCGCATCCTTAGTC--ACTGC
PC2-2                   CTTCTGCCGCCCGGTGCTGCTCGTCGCCTTTGGTCCTCG-----TCCTCAATCACACCGC
PC2-3                   CTTCTGCCGCCCGGTGCTGCTTGTCGCCTTTGGTCCTCG-----TCCTCAATCGCACCGC
```

```
SEGS2_Clone         TGCTGCTCCTCGCCGCTACGTCAATCAC-------------------------TGTGGTT
Cameroon_Episome    TGCTGCTCCTCGCCGCTACGTCAATCAC-------------------------TGTGGTT
Tanzania_Episome    TGCTGCTCCTCGCCGCTACGTCAATCAC-------------------------TGTGGTT
PC2-1               AGGTTTGAATGGGTTTTGAG------AT-------------------------TGTGGTC
PC2-2               TGCTGCTCCTCGCCGCTACGTCAATCACTGCAGGTTTGAATGTGTTTTGAGATTGTGGTT
PC2-3               TGCTGCTCCTCGCCGCTACGTCAATCACTGCAGGTTTGAATGTGTTTTGAGATTGTGGTT

SEGS2_Clone         TCATATGTGTGCTTTCTAAGATTTGTTAGATTTATTGATTTGGGTTTTTGAAATTTGCGG
Cameroon_Episome    TCATATGTGTGCTTTCTAAGATTTGTTAGATTTATTGATTTGGGTTTTTGAAATTTGCGG
Tanzania_Episome    TCATATGTGTGCTTTCTAAGATTTGTTAGATTTATTGATTTGGGTTTTTGAAATTTGCGG
PC2-1               TCAGATGTGTGCTTTCTAAG-TGTGTGAGATTTATTGATATGGGTTTCTGAAATTTGTGG
PC2-2               TCATATGTGTGCTTTCTAAGATTTGTGAGATTTATTGATTTGGGTTTTTGAAATTTGCGG
PC2-3               TCATATGTGTGCTTTCTAAGATTTGTGAGATTTATTGATGTGGGTTTTTGAAATTTGCGG

SEGS2_Clone         AAATGTTAAGATTTATATCAATGTGCTTGGGGTTGTATTCTTGAGATTTATTGAAAAAAC
Cameroon_Episome    AAATGTTAAGATTTATATCAATGTGCTTGGGGTTGTATTCTTGAGATTTATTGAAAAAAC
Tanzania_Episome    AAATGTTAAGATTTATATCAATGTGCTTGGGGTTGTATTCTTGAGATTTATTGAAAAAAC
PC2-1               AAATGTTAAGATTTATATCAATGTGCTTGGGGTTGTATTTCTGAGATTTATTGAAAATAC
PC2-2               AAATGCTAAGATTTATGCGAATATGCTTGGGGTTGTATTTTTGAAATTTATTTTGAAATT
PC2-3               AAATGCTAAGATTTATGCGAATATACTTGGGGTTGTATTTTTGAAATTTATTTTTAAATT

SEGS2_Clone         T-TTGAAATAAAGACTATTGTGAATTGATTGAGAGTTGTTTTAGTCAGATTTATTGAAAT
Cameroon_Episome    T-TTGAAATAAAGACTATTGTGAATTGATTGAGAGTTGTTTTAGTCAGATTTATTGAAAT
Tanzania_Episome    T-TTGAAATAAAGACTATTGTGAATTGATTGAGAGGTGTTTTAGTCAGATTTATTGAAAT
PC2-1               T-TTGAAATAAAGACTATTGTGAATGGATTGAGAGTTGTTTTAGTGAGATTTATTGAAAT
PC2-2               TGTTGAAATAAAGACTATAATGAATGAATATAGGGTTGTTTTTATGAGA-TTATTGAAAT
PC2-3               TGTTGAAATAAAGACTATAATGAATGAATATAGGGTTGTTTTTATGAGA-TTATTGAAAT

SEGS2_Clone         GGGTTTCTGAATTTTATTGAAATGGTACTGTGAGATTTGGTATGAATTTTGTTTTATTTG
Cameroon_Episome    GGGTTTCTGAATTTTATTGAAATGGTACTGTGAGATTTGGTATGAATTTTGTTTTATTTG
Tanzania_Episome    GGGTTTCTGAATTTTATTGAAATGGTACTGTGAGATTTGGTATGAATTTTGTTTTATTTG
PC2-1               GTGTTTCTGAATTTTATTGAAATGCTAATGTGAGATTTTGTATGAATTTTGTTTT-TTTG
PC2-2               AGGTTTATGAATTTTATTGAAATGTTAATGTGAGATT-----------------------
PC2-3               AGGTTTATGAATTTTATTGAAATGTTAATGTGAGATT-----------------------

SEGS2_Clone         TTGGGATTATGAGGTAATGGGGTTCGGGTTGTTTCGTGTAGTAAATGGATAATGGTAAAA
Cameroon_Episome    TTGGGATTATGAGGTAATGGGGTTCGGGTTGTTTCGTGTAGTAAATGGATAATGGTAAA-
Tanzania_Episome    TTGGGATTATGAGGTAATGGGGTTCGGGTTGTTTCGTGTAGTAAATGGATAATGGTAAA-
PC2-1               TTGGGATTATGAGTTAATTGGGTTCGGGTTGTTTCGTGTAGTAAATGGATAATGGTAAA-
PC2-2               ------------------------------------------------------------
PC2-3               ------------------------------------------------------------

SEGS2_Clone         --------------CGGGTTTAGGACAGATAGGGGTAGTGAAATCCAATTCCTAAACAGG
Cameroon_Episome    --------------CGGGTTTAGGACAGATAGGGGTAGTGAAATCCAATTCCTAAACAGG
Tanzania_Episome    --------------CGGGTGTAGGACAGATAGGGGTAGTGAAATCCAATTCCTAAACAGG
PC2-1               TGGATAATGGTAAACGGGTTTAGGATAGATAGGGGCAGTGAAATCCAATTCCTAAACAGG
PC2-2               ------------------------------------------------------------
PC2-3               ------------------------------------------------------------

SEGS2_Clone         GTTGGGATGGGTTTGGGTTTGGATAGTGTATTTATAAAGG-ATTCGGGTACTTAAAATTT
Cameroon_Episome    GTTGGGATGGGTTTGGGTTTGGATAGTGTATTTATAAAGG-ATTCGGGTACTTAAAATTT
Tanzania_Episome    GTTGGGATGGGTTTGGGTTTGGATAGTGTATTTATAAAGG-ATTCGGGTACTTAAAATTT
PC2-1               TTTGGGATGGGTTTGG------ATAGTGTATTTATAAAGGGATTCGGGTACTTAAAATTT
PC2-2               ------------------------------------------------------------
PC2-3               ------------------------------------------------------------

SEGS2_Clone         -CGATGGTATCCTACCCAGTACCATCCCTAATTAGAGCTTATTAGCGACCAATTTGCAAG
Cameroon_Episome    -CGATGGTATCCTACCCAGTACCATCCCTAATTAGAGCTTATTAGCGACCAATTTGCAAG
Tanzania_Episome    -CGATGGTATCCTACCCAGTACCATCCCTAATTAGAGCTTATTAGCGACCAATTTGCAAG
PC2-1               TCGATGGTATCCTACCCGGTACCATCCCTAATTAGAGATTATTAGCCACCAATTTGCAAG
PC2-2               ------------------------------------------------------------
PC2-3               ------------------------------------------------------------
```

```
SEGS2_Clone             TAACCACTCTGCTGATGATAT-ACATATATATTTAAAAGAATTAGGCATTTTTTGCTTCC
Cameroon_Episome        TAACCACTCTGCTGATGATAT-ACATATATATT-AAAAGAAT-AG-CATT
Tanzania_Episome        TAACCACTCTGCTGATGATAT-ACATATATATTTAAAAGAGTTAGGCATCTTTTGCTTCC
PC2-1                   TAACCACTCTACTGATGATGTTACATATATATTTAAAAGAATTAGGTATGTTTTGCTTCC
PC2-2                   ------------------------------------------------------------
PC2-3                   ------------------------------------------------------------

SEGS2_Clone             AATTTTGAGCCCCGTTTAAGAATTGCAATTGAAACTAAACTCCTAGCTCTTTGATTTTTA
Plant Virion                                                      CTCGCTCTTTGATTTT-A
Whitefly Virion                                                   CT-GCTCTCCGA-TTTTA
Whitefly_Episome                                                  CTAGCTCTTTGATTTT-A
Cameroon_Episome                                                  CTAGCTCTTTGATTTT-A
Tanzania_Episome        AATTTTGAGCCCCGTTTAAGAATTGCAATTGAAACTAAACTCCTAGCTCTTTGATTTTTA
PC2-1                   AATTTTTAGCCTCGTTTAAGAATTGCAATTGAAACTAAACTCCTAGCTCTTTGATTTTTA
PC2-2                   ------------------------------------------------------------
PC2-3                   ------------------------------------------------------------

SEGS2_Clone             TGAATTTAACTTGAAATCAAGTGTTGAATTTGTATGCATGTATTGTGATTTGACT-GTTC
Plant Virion            CGATTT--ACTTGAAATCAAGTGTTGAATTTGTATGCATGTATTGTGATTTGACTCGTTC
Whitefly Virion         TG-ATTT-ACTTGAAATCAAGTGTTGAATTTGTATGCATGTATTGTGATTTGACT-GTTC
Whitefly_Episome        TG-ATTTAACTTGAAATCAAGTGTTGAATTTGTATGCATGTATTGTGATTTGACT-GTTC
Cameroon_Episome        TG-ATTTAACTTGAAATCAAGTGTTGAATTTGTATGCATGTATTGTGATTTGACT-GTTC
Tanzania_Episome        TGAATTTAACTTGAAATCAAGTGTTGAATTTGTATGCATGTATTGTGATTTGACT-GTTC
PC2-1                   TGAATGTAACTTGAAATCAAGTGTTGTATTTGTATGCATGTATTGTGATTTGGCT-GTTC
PC2-2                   ------------------------------------------------------------
PC2-3                   ------------------------------------------------------------

SEGS2_Clone             TGTGTGCAAGTGAGATTTGTTAAACCGCTGGTTCTCTATTTTGTTTCCGATGTGCTGAGA
Plant Virion            TGTGTGCAAGTGAGATTTGTTAAACCGCTGGTTCTCTATTTTGTTTCCGATGTGCTGAGA
Whitefly Virion         TGTGTGCAAGTGAGATTTGTTAAACCGCTGGTTCTCTATTTTGTTTCCGATGTGCTGAGA
Whitefly_Episome        TGTGTGCAAGTGAGATTTGTTAAACCGCTGGTTCTCTATTTTGTTTCCGATGTGCTGAGA
Cameroon_Episome        TGTGTGCAAGTGAGATTTGTTAAACCGCTGGTTCTCTATTTTGTTTCCGATGTGCTGAGA
Tanzania_Episome        TGTGTGCAAGTGAGATTTGTTAAACCGCTGGTTCTCTATTTTGTTTCCGATGTGCTGAGA
PC2-1                   TGTGTGCAAGTGAGATTTGTTAAACCGCTGGTTCTCTATTTTGTTTCCGATGTGCTGAGA
PC2-2                   ------------------------------------------------------------
PC2-3                   ------------------------------------------------------------

SEGS2_Clone             TCTGTATATATGAGTTGAGAAGCAAATGATAGACGTGTTATTTATATCCTAGGATC
Plant Virion            TCTGTATATATGAGTTGAGAAGCAAATGATAGACGTGTTATTTATATCCTAGGATC
Whitefly Virion         TCTGTATATATGAGTTGAGAAGCAAATGATAGACGTGTTATTTATATCCTAGGATC
Whitefly_Episome        TCTGTATATATGAGTTGAGAAGCAAATGATAGACGTGTTATTTATATCCTAGGATC
Cameroon_Episome        TCTGTATATATGAGTTGAGAAGCAAATGATAGACGTGTTATTTATATCCTAGGATC
Tanzania_Episome        TCTGTATATATGAGTTGAGAAGCAAATGATAGACGTGTTATTTATATCCTAGGATC
PC2-1                   TCTGTATATATGAGTTGAGAAGCAAATGATAGACGTGTTA----------------
PC2-2                   --------------------------------------------------------
PC2-3                   --------------------------------------------------------
                                                       gacgtgttatttatatcctaggatcc
```

Supplementary Table 1. SEGS sequences in the cassava reference genome

SEGS-1

	Chromosome or Scaffold	Genome coordinates†		Compared to SEGS-1 clone			Repeat arrangement*	Overlap/gap size (bp)	M. esculenta transcript number		Closest Arabidopsis gene(s)	Function	Position relative to gene
				SEGS coordinates	E value	% Identity			*v6.1*	*v4.1*			
**FC1**	11	11:5691052..5692053		1-1007	0.0E+00	99.3			Manes.11G058400			Unknown	central (exon-intron-exon)
**PC1-1**	2	2:29144022..29144381	A	84-442	4.2E-100	83.2	B-A, tandem w/gap	43					
		2:29144424..29144628	B	9-214	1.6E-48	80.6							
**PC1-2**	2	2:19154829..19155072	A	16-269	6.2E-41	74.4	A-B, overlap	10	Manes.02G204400			Unknown	intron-exon-intron (antisense)
		2:19154606..19154839	B	79-354	3.5E-25	67.8							
**PC1-3**	4	4:24833006..24833285		84-396	2.2E-40	71.4			Manes.04G117100	4.1_027212m.g	AT1G15440	Ribosome biogenesis, PWP2	downstream
**PC1-4**	5	5:982124..983357	A	79-333	2.2E-78	85.9	B-A, overlap	107	Manes.05G014400		AT2G47300	RNA processing, RNase P	upstream, no UTR
		5:982015..982231	B	39-267	3.2E-38	71.9							
**PC1-5**	8	8:244125..244329		127-341	1.6E-67	86.1							
**PC1-6**	9	9:20075789..20076074		1-332	2.2E-78	79.0							
**PC1-7**	9	9:3164516..3164741		762-986	5.5E-67	86.4							
**PC1-8**	10	10:4516286..4517350	A	79-484	5.1E-118	84.1	B-A, tandem w/gap	95	Manes.10G047000	4.1_020775m.g	AT4G14145	Unknown	5'UTR/intron
		10:4516098..4516381	B	1-256	2.3E-65	79.3							
**PC1-9**	10	10:6272535..6272888		79-428	4.8E-93	81.9			Manes.10G055400			Unknown	exon (antisense)
**PC1-10**	10	10:20774488..20774770	A	84-354	5.1E-42	73.9	B-A, tandem w/gap	6					
		10:20774764..20775011	B	15-269	2.2E-40	75.4							
**PC1-11**	11	11:1151778..1152155		107-484	2.0E-138	89.1							
**PC1-12**	11	11:5687764..5687993		759-988	1.4E-93	93.0			Manes.11G058300	4.1_011981m.g	AT1G52530	DNA damage checkpoint, Hus1	5' intron
**PC1-13**	11	11:14208924..14209325	A	79-484	1.7E-130	85.8	B-A, overlap	96	Manes.11G092600	4.1_004665m.g	AT3G45890	Unknown	5'UTR/intron
		11:14209229..14209499	B	19-256	1.1E-56	77.3							
**PC1-14**	13	13:6863162..6863617		10-490	1.8E-79	71.3							
**PC1-15**	14	14:4626881..4627145	A	94-332	4.2E-43	74.5	B-A, overlap	94					
		14:4626733..4626975	B	25-268	8.1E-27	68.9							
**PC1-16**	15	15:1479690..1479952	A	92-364	2.0E-72	81.9	B-A, overlap	101	asmbl_184.cassavav61_pasa6				exon-intron
		15:1479536..1479791	B	6-266	6.66E-28	68.5							
**PC1-17**	02395	02395:2552..3025	A	79-333	2.2E-78	85.9	B-A, overlap	109					
		02395:2432..2659	B	24-267	2.7E-39	71.6							

†Sequences that are adjacent or overlap and classified as part of the same partial copy are boxed.
*Diagram of repeat arrangements

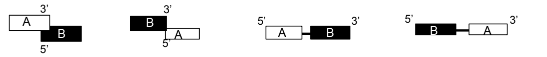

A-B, overlap   B-A, overlap   A-B, tandem w/gap   B-A, tandem w/gap

SEGS-2

	Chromosome	Scaffold*		Compared to SEGS-2 clone†			Repeat arrangement#	Overlap/gap size	M. esculenta transcript number		Closest Arabidopsis gene(s)	Function	Position relative to gene
				SEGS coordinates	E value	% Identity			*v6.1*	*v4.1*			
**PC2-1**	13	13:9112651..9113700		130-1181	0	84.1			Manes.13G073000	4.1_002333m.g	AT1G32490	RNA processing, RNA helicase	5'UTR/intron
**PC2-2**	13	13:9106290..9106936	A	37-657	0	87.8	A-B, tandem w/gap	4					
		13:9106942..9107311	B	492-873	4.1E-44	69.6							
**PC2-3**	13	13:9068208..9068836	A	56-657	0	88.7	A-B-C, tandem w/gap	5	Manes.13G072800	4.1_030164m.g	AT2G32630	PPR repeat	5'UTR/intron
		13:9068841..9069207	B	873-1142	1.75E-61	78.4		2652					
		13:9071488..9071775	C	492-873	1.75E-42	69.1							
PC2-4	1	1:22365968..22366336	A	484-877	1.0E-70	75.8	B-A, tandem w/gap	9	Manes.01G100100	4.1_017257m.g	AT5G15750	Ribosomal protein, S4	5'UTR/intron
		1:22365574..22365959	B	282-633	2.3E-66	74.3							
PC2-5	1	1:1546394..1546811		154-619	2.6E-59	71.7			Manes.01G008900	4.1_009844m.g	AT2G44150, AT3G59960	histone lysine methyltransferase	5'UTR/intron
PC2-6	1	1:2689198..2689425	A	442-672	2.6E-59	83.2							
		1:2688863..2689211	B	486-873	4.1E-44	69.8							
PC2-7	1	1:32632875..32633180	A	213-526	1.9E-48	71.1	B-A, tandem w/gap	80	Manes.01G249700	4.1_014995m.g	AT3G61620	RNA processing, 3'-5' exoribonuclease	5'UTR/intron
		1:32632477..32632795	B	500-833	5.0E-43	72.7							
PC2-8	1	1:23944969..23945179		264-488	1.4E-37	76.9				4.1_005840m.g	AT2G03820	Nonsense-mediated mRNA decay, NMD3	5'UTR/intron
PC2-9	2	2:31298773..31299279	A	172-658	2.6E-97	73.7	A-B, tandem w/gap	14	Manes.02G223500	4.1_002733m.g	AT2G40360	rRNA processing, transducin/WD40	5'UTR-ORF/intron
		2:31299293..31299660	B	503-870	1.4E-62	75							
PC2-10	2	2:20854673..20855009	A	131-658	3.8E-95	74.9	A-B, tandem w/gap	1	Manes.02G208300	4.1_017134m.g	**AT5G49210**	Unknown	upstream, no UTR
		2:20855009..20855527	B	486-833	6.1E-61	76.6							
PC2-11	2	2:22802555..22802947		264-644	5.4E-68	73.6			Manes.02G211700.1 or .2	4.1_017611m.g	AT5G37055	Chromatin remodeling, SWC6	5'UTR/intron or ORF/intron
	2	2:19104644..19105135		435-873	5.4E-49	70.0			Manes.02G204000		AT4G00695, AT1G80245	Spindle pole body component, SBP	5'UTR/intron
PC2-12	2	2:20775917..20776135		435-672	2.8E-27	72.3							
PC2-13	2	2:19291521..19291760		655-888	5.0E-24	71			Manes.02G205100.1 or .2	4.1_021797m.g	AT1G72050	Transcription factor TFIIIA	5'UTR/intron
PC2-14	3	3:2604337..2604878		115-655	4.1E-101	73.2			Manes.03G032800	4.1_020066m.g	**AT4G29660**	Unknown	5'UTR/intron
PC2-15	3	3:3943697..3944115	A	264-658	1.0E-89	77.2	A-B, tandem w/gap	1266	Manes.03G046800	4.1_006456m.g	AT5G26030	Ferrochelatase 2; FC2	5'UTR/intron
		3:3945381..3945509	B	669-872	2.6E-21	70.6							
PC2-16	3	3:17901878..17902236	A	264-650	8.0E-66	73.7	A-B, tandem w/gap	8	Manes.03G102500	4.1_012887m.g	AT3G24315	Protein transport, Sec20	5'UTR/intron
		3:17901520..17901870	B	492-873	4.7E-37	68.8							

PC2-17	3	3:22798352..22798654	A	368-658	1.8E-63	78.5	A-B, overlap	2	Manes.03G131800	4.1_017121m.g	AT1G05970	RNA binding	5'UTR/intron
		3:22798352..22798655	B	487-771	1.1E-38	76.1							
PC2-18	4	4:24555558..24555940	A	264-658	1.3E-94	78.3	A-B, tandem w/gap	1	Manes.04G111700	4.1_006233m.g	**AT1G48900**, AT5G49500, AT1G15310	Signal recognition particle SRP54	5'UTR/intron
		4:24555941..24556355	B	486-873	5.7E-74	77							
PC2-19	4	4:7561995..7562555	A	130-658	6.1E-80	73	A-B, overlap	34	Manes.04G049800	4.1_017759m.g	AT2G44860	Ribosomal protein L24e	5'UTR/intron
		4:7562521..7562761	B	454-713	3.4E-26	71.8							
PC2-20	4	4:22397356..22397790	A	173-657	3.2E-75	73.1	A-B, overlap	33	Manes.04G086400	4.1_010171m.g	AT2G21440	RNA binding; splicing	5'UTR/intron
		4:22396993..22397389	B	454-876	1.8E-54	72.4							
PC2-21	4	4:27193928..27194443		131-651	2.8E-65	69			asmbl_1260.cassavav61_pasa13				
PC2-22	4	4:26989571..26990087		131-651	2.1E-60	68.5			asmbl_1226.cassavav61_pasa13				
PC2-23	4	4:6940205..6940517		279-619	7.5E-41	71.6			Manes.04G047300	4.1_009577m.g	AT3G20320	Lipid ABC transporter, permease	5'UTR/intron
PC2-24	4	4:19138707..19139027		282-626	7.5E-41	72.7			Manes.04G066600.2	4.1_032641m.g	AT1G14650	RNA processing, RNA binding	5'UTR/intron
PC2-25	5	5:27595164..27595808		156-823	1.4E-62	66.6			Manes.05G202500	4.1_016084m.g	**AT4G24770**, AT4G34110	RNA processing, polyA binding	5'UTR/intron
PC2-26	5	5:14423021..14423452	A	185-658	6.1E-61	71.5	A-B, overlap	2	Manes.05G122900	4.1_015014m.g	AT3G17590	Chromatin remodeling, SNF5	5'UTR/intron
		5:14423450..14423698	B	486-761	5.0E-24	69.1							
PC2-27	5	5:17520978..17521210		177-428	7.0E-35	72.2							
PC2-28	6	6:9615226..9615797		83-630	2.9E-96	76.4	A-B, tandem w/gap	30	Manes.06G035600	4.1_011390m.g	**AT2G01060**	Myb-like DNA binding domain	5'UTR/intron
		6:9615827..9616033		509-711	1.2E-31	75.7							
PC2-29	6	6:8854597..8854802	A	205-641	4.4E-88	74.5	A-B, tandem w/gap	17	Manes.06G033800	4.1_005351m.g	AT4G35540	Transcription factor TFIIB-related	5'UTR/intron
		6:8854819..8855266	B	492-746	4.7E-18	66.1							
PC2-30	6	6:2553485..2559945		202-658	2.3E-85	74			Manes.06G016700	4.1_020204m.g	AT1G65032	Unknown	5'UTR/intron
PC2-31	6	6:15855902..15856507		435-798	4.0E-31	65.2			Manes.06G055300	4.1_004120m.g	AT1G72550	Phenylalanine tRNA synthetase	5'UTR/intron
PC2-32	7	7:5075189..5075753	A	112-658	3.2E-115	76.6	A-B, overlap	0	Manes.07G049900	4.1_014942m.g	AT4G33690	Unknown	5'UTR/intron
		7..5075754..5076047	B	486-791	3.2E-58	77.0							
PC2-33	7	7:10375330..10375631		202-511	4.1E-44	69.9			Manes.07G067800		AT1G13870	RNAP II elongation complex, KTI12	upstream, no UTR
PC2-34	7	7:2739275..2739772		152-738	5.7E-36	66.1			Manes.07G029100	4.1_016597m.g	AT5G47320	Ribosomal protein, S19	5'UTR/intron
PC2-35	8	8:26047010..26047364		265-644	8.5E-72	76.8			Manes.08G100300	4.1_015368m.g	**AT5G64680**	Transcription, mediator	5'UTR/intron
PC2-36	8	8:7612194..7612430	A	486-712	1.9E-48	79	B-A, overlap	13					
		8:7612016..7612207	B	466-672	6.4E-35	77.3							
PC2-37	8	8:28610112..28610384		359-609	2.8E-46	74.6			Manes.08G120700	4.1_016419m.g	AT5G27990	Pre-rRNA processing, TSR2	5'UTR/intron
PC2-38	9	9:1942319..1942850	A	130-658	1.3E-69	69.5	A-B, overlap	1	Manes.09G010600	4.1_015808m.g	**AT3G57280**	DEAD-box RNA helicase; transmembrane	5'UTR/intron
		9:1942850..1943157	B	486-791	2.1E-60	77.6							
PC2-39	9	9:12687020..12687446	A	206-647	9.7E-65	71.5	B-A, overlap	23	Manes.09G077000		AT2G32170, **AT2G32160**	SAM-dependent methyltransferase	
		9:12686673..12687043	B	487-872	1.2E-44	69.8							
PC2-40	10	10:7986732..7987004		282-557	5.02E-43	71.9			asmbl_482.cassavav61_pasa19		**AT2G15970**	Plasma membrane protein	
PC2-41	10	10:10258940..10259201		509-803	5.7E-36	72.3			Manes.10G069300	4.1_003468m.g	**AT1G32230**, AT2G35510	Cell death, RCD1	intron in 5'UTR
PC2-42	10	10:9000336..9000712		282-642	1.4E-24	68.4			Manes.10G065600	4.1_006375m.g	AT5G58340, AT1G15720, AT1G06910	Myb transcription factor, telomeric repeat binding protein	5'UTR/intron
PC2-43	11	20262512..20262927	A	264-658	2.4E-91	78.3	A-B, tandem w/gap	104	Manes.11G109500			Rab5-interacting protein (Rab5ip)	5'UTR/intron
		20263031..20263265	B	636-874	3.0E-33	74.2							
PC2-44	11	11:437662..438356		114-854	3.6E-89	71			Manes.11G003400	4.1_013005m.g	**AT1G63780**	Ribosomal RNA processing, IMP4	5'UTR/intron
PC2-45	11	11:20382103..20382477		286-632	1.8E-61	74.1			Manes.11G110200	4.1_001526m.g	**AT1G30240**	Unknown	5'UTR/intron
PC2-46	11	11:25385527..25385976		435-656	3.2E-39	77.5			Manes.11G141200	4.1_024762m.g	AT5G60335	Thioesterase superfamily	upstream, no UTR
PC2-47	12	12:4961272..4961805		131-656	2.0E-111	75.7			Manes.12G056300		**AT2G30280**	RNA-directed DNA methylation, RDM4	5'UTR/intron
PC2-48	12	12:10731671..10732124		213-657	1.87E-105	77.2							
PC2-49	12	12:8908559..8908755		213-428	1.0E-57								
PC2-50	12	12:11480337..11480650		284-658	3.9E-38	69.6			Manes.12G086400	4.1_013120m.g	AT4G28510, AT1G03860	Prohibitin	5'UTR/intron
PC2-51	13	13:3880198..3880587		264-651	9.7E-46	70.0			Manes.13G040900			Unknown	5'UTR/intron
PC2-52	14	14:24077737..24078422		143-810	4.7E-75	69.2							
PC2-53	14	14:19412662..19413134		264-743	2.3E-66	70.7			Manes.14G159700	4.1_007479m.g		Unknown	5'UTR/intron
PC2-54	14	14:19411243..19411612		264-611	1.1E-38	70.7							
PC2-55	14	14:13162957..13163180		177-423	4.7E-37	73.7							
PC2-56	14	14:12991342..12991538		203-423	1.3E-31	73.8			Manes.14G142600		AT1G08845	Mitochondrial ribosomal protein L18	5'UTR
PC2-57	15	15:14367890..14368231	A	131-645	1.1E-114	75.7	A-B, tandem	0	Manes.15G172200	4.1_003945m.g	**AT1G31870**	pre-MRNA splicing factor, RES complex	5'UTR/intron
		!5:14368232..14368785	B	486-793	2.1E-60	77.8							
PC2-58	15	15:21354291..21354728	A	265-658	2.0E-73	73.6	A-B, tandem	0					
		15:21354019..21354290	B	486-782	7.0E-54	76.1							
PC2-59	17	17:3214319..3214699	A	264-658	5.4E-87	79.2	A-B, overlap	3	Manes.17G011600	4.1_011962m.g	AT1G03360	Ribosomal RNA processing 4; RRP4	upstream, no UTR
		17:3214319..3214696	B	486-801	2.8E-46	73.2							
PC2-60	17	17:5998072..5998563	A	203-672	3.2E-77	70.5	A-B, overlap	13	Manes.17G016100	4.1_018856m.g	AT1G48160	Signal recognition protein, SRP19	5'UTR/intron
		17:5998550..5998903	B	486-873	2.6E-59	73.2							
PC2-61	00651	00651:11292..11364		130-423	2.43E-15	64.9			Manes.S022400	4.1_003682m	AT3G09720	RNA processing, RNA helicase	5'UTR
